# The immunobiology of herpes simplex virus encephalitis and post-viral autoimmunity

**DOI:** 10.1093/brain/awad419

**Published:** 2023-12-13

**Authors:** Jonathan Cleaver, Katie Jeffery, Paul Klenerman, Ming Lim, Lahiru Handunnetthi, Sarosh R Irani, Adam Handel

**Affiliations:** Oxford Autoimmune Neurology Group, Nuffield Department of Clinical Neurosciences, University of Oxford, Oxford, OX3 9DU, UK; Department of Neurology, John Radcliffe Hospital, Oxford University Hospitals, Oxford, OX3 9DU, UK; Department of Microbiology, Oxford University Hospitals NHS Foundation Trust, Oxford, OX3 9DU, UK; Radcliffe Department of Medicine, University of Oxford, Oxford, OX3 9DU, UK; Peter Medawar Building for Pathogen Research, University of Oxford, Oxford, OX1 3SY, UK; Translational Gastroenterology Unit, Nuffield Department of Medicine, University of Oxford, Oxford, OX3 9DU, UK; Children’s Neurosciences, Evelina London Children’s Hospital at Guy’s and St Thomas’ NHS Foundation Trust, London, SE1 7EH, UK; Department Women and Children’s Health, School of Life Course Sciences, King’s College London, London, WC2R 2LS, UK; Oxford Autoimmune Neurology Group, Nuffield Department of Clinical Neurosciences, University of Oxford, Oxford, OX3 9DU, UK; Wellcome Centre for Human Genetics, University of Oxford, Oxford, OX3 7BN, UK; Oxford Autoimmune Neurology Group, Nuffield Department of Clinical Neurosciences, University of Oxford, Oxford, OX3 9DU, UK; Department of Neurology, John Radcliffe Hospital, Oxford University Hospitals, Oxford, OX3 9DU, UK; Oxford Autoimmune Neurology Group, Nuffield Department of Clinical Neurosciences, University of Oxford, Oxford, OX3 9DU, UK; Department of Neurology, John Radcliffe Hospital, Oxford University Hospitals, Oxford, OX3 9DU, UK

**Keywords:** autoimmune encephalitis, herpes simplex virus encephalitis, immunology, immunotherapeutics, viral encephalitis

## Abstract

Herpes simplex virus encephalitis (HSE) is the leading cause of non-epidemic encephalitis in the developed world and, despite antiviral therapy, mortality and morbidity is high. The emergence of post-HSE autoimmune encephalitis reveals a new immunological paradigm in autoantibody-mediated disease. A reductionist evaluation of the immunobiological mechanisms in HSE is crucial to dissect the origins of post-viral autoimmunity and supply rational approaches to the selection of immunotherapeutics.

Herein, we review the latest evidence behind the phenotypic progression and underlying immunobiology of HSE including the cytokine/chemokine environment, the role of pathogen-recognition receptors, T- and B-cell immunity and relevant inborn errors of immunity. Second, we provide a contemporary review of published patients with post-HSE autoimmune encephalitis from a combined cohort of 110 patients. Third, we integrate novel mechanisms of autoimmunization in deep cervical lymph nodes to explore hypotheses around post-HSE autoimmune encephalitis and challenge these against mechanisms of molecular mimicry and others. Finally, we explore translational concepts where neuroglial surface autoantibodies have been observed with other neuroinfectious diseases and those that generate brain damage including traumatic brain injury, ischaemic stroke and neurodegenerative disease.

Overall, the clinical and immunological landscape of HSE is an important and evolving field, from which precision immunotherapeutics could soon emerge.

## Teaser

Cleaver *et al.* review the clinical presentation and immunobiology of brain disease caused by the herpes simplex virus, and discuss how relapses can be caused by immune reactions against neuronal proteins.

## Introduction

Neurotropic infections remain the leading causes of encephalitis worldwide.^1,2^ The most common epidemic global aetiology is Japanese encephalitis virus (JEV) responsible for 68 000 cases per year and is found throughout South and Southeast Asia.^3^ Herpes simplex virus encephalitis (HSE) is the leading cause of sporadic fatal encephalitis with a global incidence of 1 in 250 000 to 1 in 500 000 per year.^4,5^ Without treatment, HSE has a mortality rate of 70%, which is reduced to 10%–25% with aciclovir.^6^ Despite optimal therapy, around half of all survivors are left with significant neurological disability. Recurrence of a neurological syndrome is common, occurring in 5%–27% of patients according to the most recent studies, predominantly within the first 2 months of disease onset and often without detection of the herpes simplex virus (HSV).^7-9^

Population-based studies highlight the archetypal HSE as a monophasic disease associated with an acute fulminant inflammatory brain reaction but readmission following the initial acute admission is common.^4,10,11^ Although the true burden of relapsing events after acute HSE is incompletely understood, in some cases, viral reactivation or autoimmune encephalitis (AE) occurs. The landmark placebo-controlled study demonstrated that 6 months of viral suppressive therapy following completion of parenteral therapy in neonatal HSE improved neurodevelopmental outcomes.^12^ This suggests subclinical reactivation of HSV occurs in the brain after resolution of the acute infection and this reactivation contributes to the neurologic injury that infants with neonatal-onset HSE experience. In exceptional circumstances, chronic brain inflammation following HSE has been documented but current evidence is limited to predominantly older neuropathological reports thus requiring substantiation by way of larger population-based studies.^13-15^

The emerging clinical phenotype of post-HSE AE is typically characterized by a combination of new behavioural changes, encephalopathy, seizures and movement disorders.^16^ Post-HSE AE responds favourably to immunotherapy and is typically associated with the formation of antibodies against synaptic neuronal cell surface receptors; most frequently the GluN1 subunit of the *N*-methyl D-aspartate receptor (NMDAR).^16^ Further studies have identified antibodies directed against other neuroglial targets including GABA A receptor (GABA_A_R),^16^ contactin-associated protein-like 2 (CASPR2),^17^ leucine-rich glioma-inactivated 1 (LGI1),^18^ glial fibrillary acidic protein (GFAP)^19,20^ and dopamine 2 receptor,^21^ as well as other unknown antigenic targets.^16^

Given the disabling nature of HSE and incomplete understanding of neuroinflammation in response to the virus, in this review, we explore the innate and adaptive immunobiology at different clinical stages in the disease course. Additional focus on the phenotype and immunobiology relevant to secondary autoantibody-mediated encephalitis are reviewed followed by translational concepts within neurology and infectious diseases. Improved understanding of the temporal changes in the immune milieu during HSE offers promise for future prognostic biomarkers and novel immunotherapeutic strategies.

## Acute herpes simplex virus encephalitis

**Figure 1:**
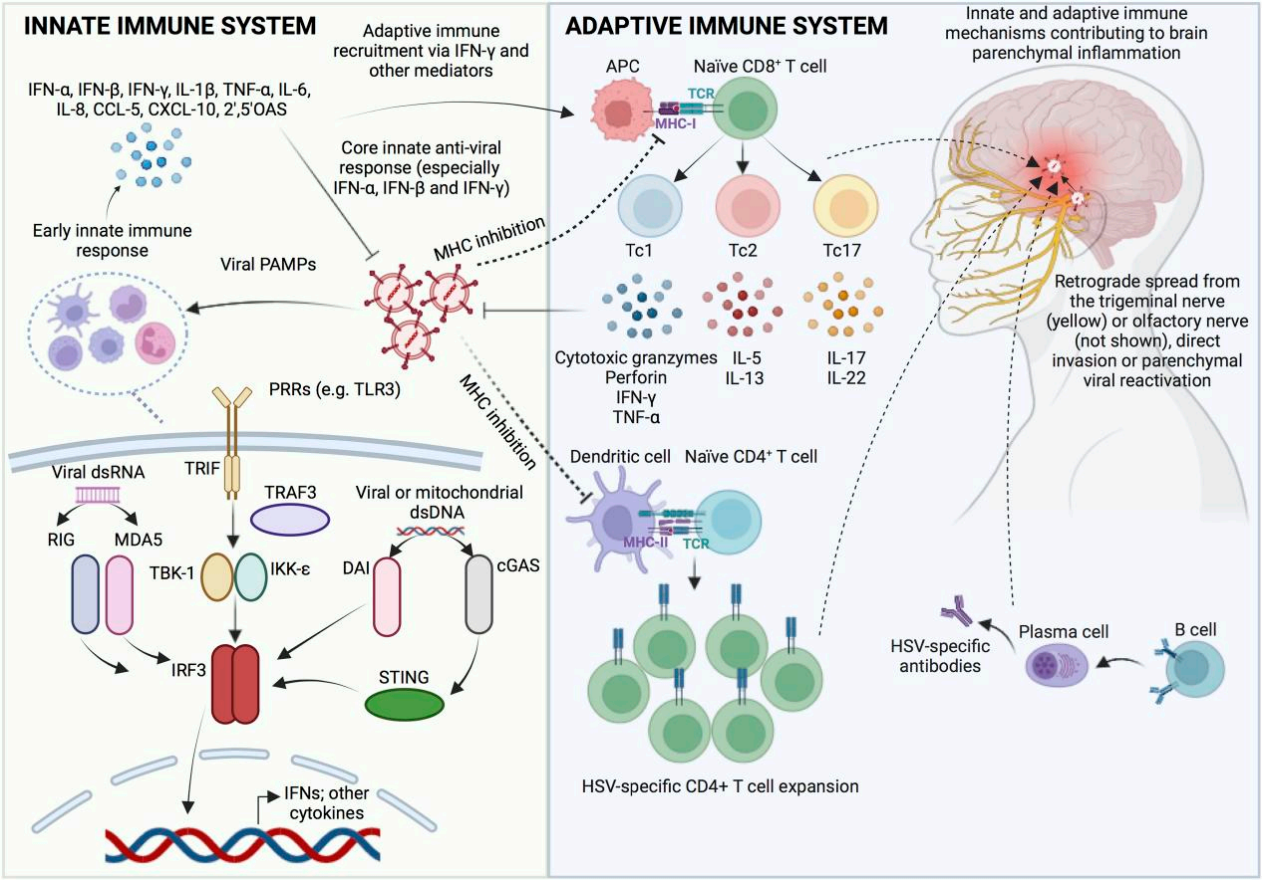
**Host immune response to HSV.** An early innate immune response, triggered by viral pathogen-associated molecular patterns (PAMPs), occurs through dimerization of the PRRs generating transcription of IFNs and other cytokines; predominantly through TLR3 and its downstream molecules. This stimulates the release of pro-inflammatory cytokines, which facilitate blood–brain barrier breakdown and recruitment of, predominantly, CD8^+^ T cells but also other arms of the adaptive immune system. To counter adaptive cell-mediated immune elaboration, HSV-1 attempts to evade host MHC expression through a variety of different mechanisms. These processes ultimately contribute to the antiviral response and local neuroinflammation. 2′,5′OAS = 2′-5′-oligoadenylate synthetase; APC = antigen-presenting cell; CCL-5 = chemokine CC motif ligand 5; cGAS = cyclic GMP-AMP synthase; CXCL10 = chemokine CXC motif ligand 10; HSV-1 = herpes simplex virus; IFNs = interferons; IL = interleukin; IRF-3 = interferon regulatory factor 3; MAG = myelin associated glycoprotein; MDA5 = melanoma differentiation-associated protein 5; MHC = major histocompatibility complex; NMHC-IIA = non-muscle myosin heavy chain-IIA; PRRs = pattern recognition receptors; RLRs = retinoic acid inducible gene-I (RIG-I)-like receptors; STING = stimulator of interferon genes; TLRs = toll-like receptors; TNF-α = tumour necrosis factor alpha.

HSV type 1 (HSV-1) is a member of the Herpesviridae family of DNA viruses. HSV-1 entry to the host is initiated through its glycoproteins structures, which facilitate host receptor binding and receptor-mediated endocytosis (Fig. 1). An estimated 80%–85% of young adults are seropositive for HSV-1^22,23^ and it is the most common viral cause of sporadic fatal encephalitis worldwide affecting children and adults.^24^ Neonatal HSE is almost invariably caused by HSV-2 presenting similarly to HSV-1 CNS disease in adults and children. HSE onset can be triggered by primary infection or, more commonly in adults, reactivation of latent HSV-1. Stress is a known trigger for viral reactivation with some evidence for the role of catecholamine and glucocorticoid downstream pathway activation in facilitating viral escape.^25-28^ Despite this, the molecular processes driving viral reactivation are incompletely understood. The detailed mechanisms of primary HSV-1 infection, viral reactivation and mucosal immunity are beyond the scope of this article and have been reviewed previously.^29-31^

### Clinical observations

Clinical manifestations of acute HSE are typically severe and include prodromal symptoms followed by persistent fevers, headache, encephalopathy, personality/behavioural changes and seizures.^10^ CT brain can be normal acutely but an MRI is abnormal in almost all cases and often reveals asymmetric hyperintense lesions with oedema, diffusion restriction, contrast enhancement and, not uncommonly, petechial haemorrhages or haemorrhagic necrosis.^10^ CSF usually reveals a lymphocytic pleocytosis—although a neutrophil predominance can be found early in the disease course—mild to moderately raised protein and the red cell count may be elevated.^32^ Certain neuroimaging features are associated with poorer outcomes, including involvement of more than three lobar brain regions or left-sided thalamic diffusion signal change.^33,34^ Disability in survivors is significant, with 48% classed as moderate to severely disabled and with frequent residual cognitive impairments.^35^ Given the temporal lobe predominance of HSE, verbal memory is most affected, including name and object recall, listening and recalling spoken information.^36,37^ Other cognitive difficulties include impairment of processing speed, concentration and executive function.^38^ Further information on the clinical manifestations of HSE have been previously reviewed in detail.^39-41^

Patients with HSE typically exhibit pro-inflammatory CNS cytokine profiles associated with neuronal cell death and which can vary by the clinical trajectory (Table 1).^42-46^ This suggests that the host immune response is likely to have deleterious effects. Specifically, acute CSF interferon (IFN)-γ, monocyte chemoattractant protein (MCP)-1 and IL-6 are associated with poorer outcomes.^42^ Overall, although the initial inflammatory response is directed towards suppressing viral pathology, it can have deleterious effects on the outcome of patients with HSE.

**Table 1:** **CSF cytokine and cell surface markers associated with different HSE disease stages as observed** ^42-46^

ACUTE HSE		
CSF markers	Functional subgroups	Physiologic effect
Interferons	↑ IFN-γ	Anti-viral immunity
Interleukins	↑ IL-6	Increase B cell activation and differentiation
	↑ IL-8	Granulocyte chemoattractant
	↑ IL-10	Anti-inflammatory properties including inhibition of IFN-γ
Tumour necrosis factors	↑ TNFR1, ↑ TNF-α	Pro-inflammatory pyrogenic, non-specific immunity, BBB breakdown
Chemokines	↑ MCP-1	Monocyte and T cell chemoattractant

CHRONIC HSE		
CSF markers	Functional subgroups	Physiologic effect
Interleukins	↑ sIL-2R	Role in tolerance; activated T-cell marker
Tumour necrosis factors	↑ TNF-α	Pro-inflammatory pyrogenic, non-specific immunity, BBB breakdown
CDs	↑ sCD8 antigen	Cytotoxic T-cell signalling

RELAPSING HSE SECONDARY TO AUTOIMMUNE ENCEPHALITIS		
CSF markers	Functional subgroups	Physiologic effect
Interferons	↑ IFN-α	Anti-viral immunity
Chemokines	↑ CXCL9, ↑ CXCL10	Th1-mediated chemokines
	↑ CXCL13, ↑ CCL19, ↑ APRIL	B cell-mediated chemokines including markers of germinal centre activity

APRIL = a proliferation inducing ligand; BBB = blood–brain barrier; CCL = chemokine CC motif ligand 5; CD = cluster of differentiation; CXCL = C-X-C motif chemokine ligand; HSE = herpes simplex virus encephalitis; IFN = interferon; IL = interleukin; MCP = monocyte chemoattractant protein; sIL-2R = soluble interleukin-2 receptor; sCD8 = soluble CD8; Th1 = T-helper 1; TNF = tumour necrosis factor; TNFR = tumour necrosis factor receptor.

Although broad immunosuppression is not associated with an increased incidence of HSE, sporadic monogenic inborn errors of immunity have been suggested to occur in ∼5% of HSE cases.^47^ Mutations associated with HSE most often impair function of the innate immune system, with the most commonly reported defects affecting the Toll-like receptor 3 (*TLR3*) gene or its downstream signalling components (*UNC93B1*, *TRIF*, *TRAF3*, *TBK1* or *IRF3*).^47^ Under normal circumstances, activation of this pathway facilitates transcription of *IFN* genes to protect the host against HSV-1 (refer to the ‘Relapsing herpes simplex virus encephalitis’ section). Current evidence supports genetic testing for paediatric patients—especially those with recurrent proven HSE^48^—although adult patients are also being identified.^49^

### Innate immunity

The initial biological mechanisms through which HSV-1 enters the brain remains uncertain and is predominantly supported by experimental animal studies. Trans-synaptic retrograde spread from the trigeminal neurons to the meninges and then to the frontal and mesiotemporal lobes is plausible,^50^ although countered by the relative rarity of HSE in other regions also connected with the trigeminal nerve, such as brainstem encephalitis.^51^ The olfactory tract is another potential entry portal. This has direct connections to the ipsilateral frontal and mesiotemporal lobes with potential for contralateral spread via the anterior commissure.^52,53^ Interestingly, recurrent forebrain^23,47,54-56^ and recurrent brainstem HSE^57^ have been associated with different monogenic mutations suggesting gene expression could be important for regional-specific viral tropism within the CNS. This is discussed further in the ‘Relapsing herpes simplex virus encephalitis secondary to viral reactivation’ section. Viral entry mechanisms and host immune responses are illustrated in Fig. 1.

The blood–brain barrier (BBB) is a network of confluent endothelial cells with interlinking tight junction proteins, pericytes and astrocytes that regulate foreign pathogen and host immune trafficking to the brain. In HSE, alterations in trafficking across the BBB is complex and incompletely defined. However, failure of BBB function as defined by brain imaging biomarkers correlate with poorer outcomes.^58^ Moreover, murine models of HSE have been used to explore mechanisms behind BBB permeability, CNS transmigration of the immune milieu and consequential effects on viral replication within the brain.^59,60^ Indeed, an upregulated intraparenchymal immune response was found within the brains of HSV-1 infected mice.^59^ Moreover, chemokines CXCL1 and CCL2 are released following HSV-1 CNS infiltration with Ccr2 (CCL2 receptor)-deficient mice exhibiting reduced monocyte ingress, uncontrolled viral replication and poorer outcomes.^60^ Conversely, Cxcr2 (CXCL1 receptor)-deficient mice displayed lower neutrophil transmigration, improved BBB integrity and had better outcomes despite a similar viral load. Despite this evidence, in human patients with HSE, a lymphocytic pleocytosis is often observed. Further work is required to explore BBB trafficking of specific immune subpopulations longitudinally across the disease illness.

Viral reactivation within the parenchyma or direct CNS invasion through the aforementioned processes are postulated mechanisms of CNS disease onset.^39^ Upon reaching the CNS, HSV replicates within cells, causing cell swelling, plasma membrane fragmentation and nuclei degradation, with the formation of polynuclear giant cells. The initial mechanism through which this occurs is via host recognition of viral pathogen associated molecular patterns (PAMPs). PAMPs are evolutionarily conserved molecular structures found in microorganisms, which are recognised by a limited number of germ line-encoded pattern recognition receptors (PRRs) found on innate immune cells. PRRs are pathogen-derived nucleic acid sensors that provide the host with vital innate immune surveillance machinery in its defence against CNS HSV-1 infection. TLR3 and its downstream mechanisms have been the most studied in HSE, driven by the increased susceptibility to HSE associated with mutations in this pathway.^61,62^ Moreover, supportive experimental work has identified high susceptibility to HSV-1 infection in TLR3-deficient neurons and oligodendrocytes cultured from induced pluripotent stem cells (iPSCs).^63^ Upon activation by HSV-1 double-stranded RNA (dsRNA) PAMPs, TLR3 dimerizes causing activation of TRIF-dependent pathways.^64^ Subsequently, there is activation of transcription factors including interferon regulatory factor 3 (IRF3) and nuclear factor-kappa B (NF-κB).^54,61,62^ These transcription factors trigger production and release of pro-inflammatory mediators including, predominantly, IFNs but also tumour necrosis factor alpha (TNF-α), interleukin 1β (IL-1β), IL-6, IL-8, chemokine CC motif ligand 5 (CCL5), CXC motif ligand 10 (CXCL10) and 2′-5′-oligoadenylate synthetase (2′,5′OAS).^65^ Collectively, this can lead to recruitment of different leucocyte subpopulations and modulate the inflammatory environment within the brain in response to HSV-1.^65^

Microglia are innate CNS-resident immune cells that serve as early sensors of HSV infiltration to the brain. Microglial PRRs trigger the cyclic-GMP-AMP synthase-stimulator of interferon genes (cGAS-STING)-dependent pathways to aid viral clearance through leucocyte recruitment.^66^ Engagement of the cGAS-STING axis, in turn, triggers type I IFN production together with release of cytokines and chemokines including IL-1β, TNF-α, CCL2, CCL5 and CXCL10.^67^ Data from murine models support a role for microglia in response to HSV-1 CNS infection: higher CNS viral loads have been observed in cGAS-deficient versus wild-type mice, microglia co-localize with HSV-1 within the brain and microglial depletion increases susceptibility to HSV-1 CNS infection.^66,68^

There is emerging evidence for the role of three other PRRs—retinoic acid inducible gene (RIG)-I, DNA-dependent activator of interferon regulatory factors (DAI) and melanoma differentiation-associated gene 5 (MDA5)—which serve predominantly as cytosolic nucleic acid sensors. Similar to TLR3 stimulation, their interaction with pathogen-derived genetic moieties activates downstream IRF3 and NF-κB pathways to facilitate a type I IFN response. Although its role in HSE is not entirely clear, preclinical evidence has shown upregulation of DAI within microglia and astrocytes following infection with HSV-1 in cell line and murine models of HSE, with knockdown models preventing downstream pro-inflammatory cytokine release.^69^ Moreover, RIG-I can be activated by DNA viruses, which undergo transcription to RNA via RNA polymerase III. Indeed, RIG-I knockdown and transfection with siRNA directed to RIG-I attenuates TNF-α responses in microglia and astrocytes.^70^ A recent study described a child afflicted by HSE who was found to harbour a compound heterozygous variant in the GTF3A gene, which encodes for the transcription factor IIIA (TFIIIA); a moonlighting protein with protective properties against HSV through production of the RIG-I ligand, RNA5SP141.^71^ This patient (and his sister) exhibited a diminished IFN-I response to HSV-1 in isolated patient fibroblasts and GTF3A gene-edited cells.

Core antiviral innate responses to HSV-1 PAMPs are induced by IFN proinflammatory cytokine signals; most especially IFN-α, IFN-β and IFN-γ. Upon stimulation of their cognate receptor, IFNs trigger the expression of genes creating an antiviral response in infected cells.^72^ Indeed, many *in vitro* and *in vivo* studies demonstrate the importance of a robust IFN-α and -β response in reducing viral replication, spread and cytopathic effect.^73,74^ Furthermore, IFN-γ facilitates elaboration of the host's innate to the adaptive immune arm by triggering formation of MHC-I, which allows antigen presentation to HSV-specific CD8^+^ T cells. In addition, IFN-γ, with IFN-α and/or IFN-β, synergistically reduces viral replication.^75^

Although its direct role in HSE is not well understood, natural killer (NK) cells constitute an important first-line immunological defence to HSV-1 infection with patients suffering from natural killer cell deficiency disorders susceptible to more severe, recurrent and occasionally fatal infection.^76^ One observational study identified five paediatric acute HSE patients who had concomitant monogenic natural killer deficiencies with clinical clues including recurrent viral infections, lymphopaenia and significant reduction in T (CD4^+^, CD8^+^) and B cell subsets.^77^ Moreover, an adult patient with natural killer cell deficiency harboured an X-linked hemizygous germline variant in the *IL2RG* gene and exhibited a long history of severe recurrent human papillomavirus disease.^78^

Upon activation by the initial IFN response to HSV-1, natural killer cells express functional proteins, including CD16a and IFN receptors, and secrete cytokines, perforin and granzyme B to kill infected cells.^79^ In addition, prior to the establishment of HSV-1-specific adaptive immunity, immunoglobulin G (IgG) plays an important role in coating viral-infected cells for subsequent recognition and clearance by natural killer cells.^79^ This occurs through interaction of IgGFc with viral glycoprotein E (gE) to allow clearance by natural killer cells by Fc-bridged cell-mediated cytotoxicity (FcBCC). Moreover, natural killer cells may also increase their expression of their surface receptor NKG2D potentially sensitizing natural killer cells to HSV-1 infected cells.^80,81^ To counteract the host natural killer response, HSV-1 can downregulate the expression of MHC-I on host cells.^82,83^ The disruption of MHC expression by HSV-1 has also recently been shown to attenuate the activation of mucosal-associated invariant T cells, demonstrating the elaborate mechanisms by which the virus attempts to evade innate immunity (Box 1).^84^

Box 1 **Mucosal-associated invariant T cells and HSV-1 infection**

A recent discovery in the interaction between HSV-1 and the innate immune system has identified a potential novel role for mucosal-associated invariant T (MAIT) cells.^84^ MAIT cells are defined as innate-like T cells characterized by their semi-invariant αβ T cell receptor (TCR). They possess the ability to recognize bacterial, fungal and, more recently, viral pathogenic metabolites derived from riboflavin (vitamin B_2_) intermediates through interaction with the antigen-presenting molecule MHC-I-related gene protein (MR1).^85^ Moreover, MAIT cells may have a relevant emerging role in protection against neuroinflammation by preventing reactive oxidative species-induced meningeal barrier damage.^86^

In contrast to conventional T cells, MAIT cells do not possess the highly variable TCRs that undergo refinement and expansion to target an array of epitopes, but instead possess an innate ability to target a specific set of ligands.^85^ Indeed, in part due to the expression of Us3 protein, HSV-1 possesses the ability to profoundly downregulate MHC-I expression,^87^ including MR1 from the surface of antigen-presenting cells; targeting it for proteasomal degradation.^84^ In turn, this reduces MAIT TCR-dependent activation and highlights the myriad mechanisms by which HSV-1 can modulate the host's immune response through disruption in the function of MHC and MHC-like expression.^84,88,89^ Moreover, the observation that certain primary immunodeficiency disorders associated with a significant reduction in MAIT cell numbers increase susceptibility to viral infections—including HSV-1—support their role in the immunopathogenesis of primary infection.^90-93^ However, further *in vivo* work is necessary to confirm the role of MAIT cells in HSE and whether MR1 or MR1-independent mechanisms of immunomodulation are most contributory.

Evidence suggests the early innate immune responses to HSV are crucial for the host to protect against acute HSE. In particular, intact TLR3 pathways likely play a significant role in resisting CNS viral spread. Despite downregulating its defence against acquisition of HSE, it is unknown whether dysregulation in the host innate immune pathway alters the clinical severity of disease from incurring harmful neuroinflammation. Reviewing innate genotype-phenotype correlations and further reviewing longitudinal cytokine and surface markers may help clarify this further.

### Adaptive immunity

CD8^+^ T cells have a pivotal role in the host adaptive immune response against viral infection.^94,95^ Before activation of an effective CD8^+^ adaptive cellular response, the host must overcome resistance from HSV-1, which expresses an immediate-early protein, ICP47.^96^ ICP47 functions to prevent migration of antigenic peptides and causes MHC-I accumulation within the endoplasmic reticulum. Assuming host circumnavigation of viral MHC-evasion strategies, antigenic presentation by MHC-I to naïve CD8^+^ T cells promote differentiation of these cells to different CD8^+^ subsets including T cytotoxic (Tc)1, Tc2 or Tc17 cells.^97,98^ Tc1 is also known as a cytotoxic lymphocyte and has a crucial role in clearing intracellular pathogens through release of cytotoxic granzymes and perforin, whilst concomitantly secreting IFN-γ and TNF-α to consolidate the innate and adaptive immune response to intracellular pathogens.

Subsequently, CD8^+^ T cells egress from lymphoid tissue in the form of T_RM_ cells and become compartmentalized within the neural ganglia and local mucosa.^99^ The role of T_RM_ is to broadly contain infection, assist with local antigen presentation, and contribute to local inflammation and T cell activation.^100^ Within the brain, T_RM_—referred to as brain T_RM_ or bT_RM_—are compartmentalized in response to the local cytokine/chemokine milieu including IL-1, IL-6, IL-12, TNF-α and induction of MHC expression.^101,102^ Moreover, CD4^+^ T cell release of TGF-β compounds T_RM_ recruitment.^103^ Upon antigen stimulation, bT_RM_ secrete IFN-γ, TNF-α, IL-2 and IL-17, together with possessing the ability to kill infected cells via granzyme B and perforin-mediated mechanisms.^101^ Importantly, bT_RM_ may also harbour the ability to halt immune overactivation, refining the immunological cascade, through increasing their expression of the immune checkpoint programmed death 1 (PD-1).^104^ Furthermore, increased expression of MHC-I heavy chain, H-2K^b^, by CNS dendritic-like cells and macrophage-like cells has been identified during CNS infiltration of HSV-1 suggesting a role in antigen presentation to HSV-1-specific CD8^+^ T cells.^105^ However, given the parsimonious expression of MHC by neurons the precise CD8^+^ T cell effector mechanisms within the CNS requires further clarification.

CD4^+^ T cells have the ability to restrict and eliminate acute HSV infection but also regulate the host immune response.^100^ Interestingly, in the HSV-1 corneal infection model, upon activation by local dendritic cells, naïve HSV-specific CD4^+^ T cells undergo expansion and CD4^+^ T effector cells are restimulated within draining lymph nodes.^106^ The purpose of this immune arborization is thought to regulate the local deleterious effects of inflammation. Viral counter mechanisms include HSV-1-induced modification of the MHC-II processing pathway, through inhibiting expression of MHC-II invariant chains and also via gB binding HLA-DR and HLA-DM heterodimers.^107^

The role of the host T cell response in acute HSE is important, with experimental studies highlighting a spectrum from effective viral CNS clearance to a maladaptive immune reaction, which may exacerbate brain inflammation. In mouse models of HSE, CD8^+^ T cells appear to co-localize with focal necrotic brain lesions^108^ but in those with severe combined immune deficiency (SCID)—an adaptive immunodeficiency—these focal necrotic lesions were absent despite high levels of viral replication within the brain.^109^ Moreover, mortality timing was delayed in SCID mice, suggesting early phenotype severity may be linked to intact adaptive immune mechanisms. In addition, delayed expansion of CD8^+^ T cells was positively correlated with worsening disease in stress-induced HSE mouse models.^110^ Prior to infection, mice that were vaccinated with a recombinant vaccinia virus vector expressing a single cytotoxic T lymphocyte (CTL) recognition epitope of HSV-1 (gB498–505), were devoid of HSV infection within the brain and consequently did not succumb to HSE.^110^ Therefore, the T cell response to HSV infection in the CNS must balance optimal viral clearance with immune-mediated neuronal damage.

Despite secondary autoantibody formation in ∼25% of patients after HSE, understanding of the B cell response in acute HSE is limited. Observational studies have identified that patients with combined variable immunodeficiency (CVID)—a disorder producing a quantitative and qualitative reduction in antibody production and subsequently insufficient response to polysaccharide vaccines—not treated with intravenous immunoglobulins developed acute HSE suggests the humoral response may play an important role.^111^ Supporting this, B cell deficiency mouse models have an increased susceptibility to HSE.^112,113^ Following HSE, intrathecal HSV-specific IgG has been shown to remain present for years, despite a negative viral PCR.^114,115^ A more recent study analysed CSF and peripheral B cells through single cell RNA sequencing from a HSE patient demonstrated dynamic and early expansion of B cell clones with intercompartmental overlap.^116^ The role of this B cell clonal expansion remains uncertain, with further research required to understand antigenic specificity.

A potential role for peripheral germinal centre (GC) reactions in the acute HSV-associated B cell response, is provided by a rodent study of ocular herpes secondary to HSV-1, in which elevated levels of HSV-specific CD19^+^CD27^+^ memory B cells together with increased frequencies of HSV-specific switched IgG^+^CD19^+^CD27^+^ memory B cells were identified in asymptomatic animals.^117^ The emergence of data supporting the role for the meningeal lymphatics in facilitating immune cross-talk between the brain and periphery, through cervical lymph nodes, highlights a plausible mechanism by which germinal centre reactions may mediate the B cell response to HSE and potentially underlie secondary AE.^118-121^

## Chronic herpes simplex virus encephalitis: current knowledge and controversies

The evidence for chronic HSE is limited and is based around older neuropathological reports, thus its true existence as a natural progression from acute HSE remains to be determined. Yet, consistent neuropathological and radiological themes from this limited data have emerged and warrant consideration. For instance, a smouldering inflammatory response has been observed in the CNS following acute HSE.^14,15,122,123^ Typically, this process occurs in the absence of CSF viral DNA and neuroglial surface autoantibodies (NSAbs), but neuropathological evaluation can often identify the presence of HSV DNA from brain tissue. Chronic HSE has only been phenotyped to a limited extent and likely occurs on a spectrum shared with relapses secondary to AE. Current available evidence suggests that chronic HSE can manifest clinically from months to over a decade in some cases.^15^

Phenotypic presentations observed include seizures, raised intracranial pressure and psychomotor symptoms.^15^ CSF has shown to be sterile or reveal a mild lymphocytic pleocytosis, typically with the presence of unpaired oligoclonal bands and negative HSV PCR but with evidence of intrathecal HSV-specific IgG synthesis.^13-15,122-124^ Distinct from classical HSE and relapsing secondary autoantibody encephalitis, an MRI brain characteristically reveals confluent gyriform cortical and adjacent white matter enhancement with vasogenic oedema.^15,124^ Optimal management is unclear but some reports describe patients receiving repeat intravenous aciclovir, with steroid treatment, albeit with variable outcomes.^15,124^

Neuropathological findings in chronic HSE appear somewhat distinct from acute HSE, characterized predominantly by a necrotizing inflammatory process, instead demonstrating chronic granulomatous inflammation with foci of mineralization.^13,15,124^ Infiltration of CD68^+^ macrophages, multinucleated giant cells, CD3^+^ T-lymphocytes and CD138^+^ plasma cells, in the presence of low-level HSV-1 viral DNA detected by way of PCR, have been described.^13,15,124^

## Relapsing disease secondary to viral reactivation

Relapsing disease secondary to viral reactivation—proven by clinical deterioration with evidence of viral DNA or antigens within the brain or CSF in patients treated appropriately with aciclovir—has been observed in 5% of paediatric patients with fewer reported cases in adults (reviewed further in Alsweed *et al*.^125^). HSV DNA has been identified in HSE survivors for many years without clear phenotypic relapse.^14,123^ Despite this, there have been reports of proven recurrent HSE in children with relapses described up to 5 years following index presentation, which has led to the discovery of relevant monogenic inborn errors of immunity.^48,126^

Monogenic immunodeficiencies rendering the host susceptible to HSE predominantly involves inborn errors of TLR3-dependent IFN-α/β- and IFN-λ-mediated immunity.^54,127,128^ An isolated forebrain form of HSE is found in some children with errors in *TLR3* pathway genes^23,47,54,55^ or in *SNORA31*.^56^ The *SNORA31* gene encodes for the small nucleolar RNA 31 (SnoRNA31) protein and, although its role is not fully understood, it is thought to be involved in isomerization of uridine to pseudouridine in 18S ribosomal RNA (rRNA) of cortical neurons.^129^ CRISPR/CASP9-induced deletion of *SNORA31* revealed a heightened susceptibility to HSV-1 infection within neurons derived from iPSC although, importantly this study did not model mutations known to be associated with forebrain HSE.^56^ Intriguingly, an isolated brainstem HSE has been observed in association with a *DBR1* mutation encoding for debranching enzyme 1 (DBR1); a protein responsible for RNA lariat-debranching.^57^ DBR1 is preferentially expressed within the brainstem and spinal cord with a partial deficiency identified in brainstem encephalitis associated with HSV-1, influenza B and norovirus.^57^ Studies involving DBR1-deficient fibroblasts reveal increased susceptibility to HSV-1, potentially secondary to the impairment of host intrinsic cellular immune responses to HSV-1 through accumulation of RNA lariats.^130,131^ Nevertheless, the true mechanism behind different topographical HSE pathologies in distinct monogenic mutations remains elusive.

Further notable phenotypic clues to a monogenic deficiency, in addition to HSE recurrence, include other recurrent viral diseases and severe reactions to vaccines.^132^ Overall, albeit uncommon, a monogenic defect in TLR3- and IFN-pathways should be suspected, primarily in children, especially with relapsing forebrain HSE secondary to viral reactivation, whereas *DBR1* deficiencies should be sought in such cases of brainstem HSE.^128,133^

## Relapsing disease secondary to autoimmune encephalitis

Building on early reports that HSE patients may exhibit a biphasic clinical phenotype and improve with adjuvant steroid treatment, in 2012, a retrospective study led by a group in Germany discovered the presence of serum antibodies targeting the NMDAR from 13 of 44 HSE patients (30%).^134^ Two years later, several different groups observed clinical relapses from HSE cohorts in the presence of, predominantly, NMDAR autoantibodies and which frequently responded favourably to steroids.^21,135,136^ More recently, a larger Spanish prospective study and retrospective analysis identified that 27% of patients with HSE clinically relapse, despite the absence of viral recurrence identified through CSF PCR.^16^ Further prospective studies in other independent clinical cohorts are important to substantiate the true degree of autoimmune relapses after HSE.

### Review of the literature

To provide a contemporary analysis of the emerging post-HSE AE phenotype, we have comprehensively searched the literature from 2007 to March 2023 (Supplementary material) to review the clinical features, investigations, management and outcomes. The phenotypes of acute HSE, relapsing HSE secondary to AE and classical NMDAR AE are compared in Table 2.

**Table 2:** Clinical comparisons between classical HSE, post-HSE AE and classical NMDAR AE

Demographics and clinical/paraclinical features	Classical HSE^a^	Post-HSE AE	Classical NMDAR AE^b^
Median age, years (range)	All ages; bimodal distribution with ∼33% children/adolescents and ∼50% >50 years	13 (2 months–84 years)	21 (2 months–85 years)
Clinical features	Fever, headache, behavioural abnormalities, altered consciousness, seizures, focal neurological deficit	Behavioural changes, new-onset seizures, movement disorder, encephalopathy are most frequent. Autonomic dysfunction, insomnia and new focal neurological deficits are uncommon	Encephalopathy, neuropsychiatric features, movement disorder, language disorder, autonomic dysfunction, central apnoea
MRI brain scan findings	∼80%–100% abnormal revealing mesial temporal, insular, cingulate and/or orbitofrontal high signal	∼30%–40% new or progressive MRI-brain changes; necrosis and cystic lesions more likely	70%–80% normal; minority limbic high signal
CSF findings	∼90% abnormal, typically with lymphocytic pleocytosis and modestly raised protein >0.5 g/l	∼2/3 a lymphocytic pleocytosis and modestly raised protein >0.5 g/l, often with unpaired oligoclonal bands; the remainder are typically normal	80% abnormal, typically lymphocytic pleocytosis, unpaired oligoclonal bands
Outcomes	70% mortality without treatment reducing to 20% with aciclovir; ∼50% survivors have an mRS >2	∼70% have a poor outcome—more so in children ≤4 years—with an mRS >2. The remaining majority exhibit some disability	∼50% improve with first-line IT; ∼70% of non-responders improve after second line IT; 80% reaching mRS 0–2

AE = autoimmune encephalitis; HSE = herpes simplex virus encephalitis; IT = immunotherapy; mRS = modified Rankin score; NMDAR = *N*-methyl D-aspartate receptor.

^a^Summarized through references.^10,137-139^

^b^Summarized through references.^140-144^

We identified 110 patients from 32 publications with a median age of 13.5 years (range, 2 months–84 years) and a female-to-male ratio of 1.4:1. The median time from HSE to secondary AE was 30 days (range, 7–510 days). Patients were dichotomized by age into those ≤4 years versus >4 years, in line with the diverging clinical phenotype observed in the most recent observational study.^16^ The frequencies of movement disorders (*P* < 0.0001), encephalopathy (*P* = 0.0013) and insomnia (*P* = 0.0187) were all higher in patients ≤4 years.

The precise antigenic target also differed by age: NMDAR autoantibodies were found more often in patients ≤4 years (*P* = 0.0026) whereas almost one-third of those >4 years had unknown antigenic targets (*P* = 0.0021). There were no significant differences in the MRI-brain findings at AE relapse between the two groups with 23.1% and 48.3% of patients displaying new or progressive changes in patients ≤4 years and >4 years, respectively. Similarly, there were no significant differences in treatment strategies between the two groups. Despite this, outcome data significantly diverged with patients ≤4 years suffering more significant disability overall [modified Rankin score (mRS) >2; *P* < 0.0001].

### Additional clinical observations

Post-HSE AE is typically defined as a deterioration in neurological symptoms for >24 h and in the absence of CSF HSV-1 DNA with exclusion of an alternative medical cause.^16^ Neuropsychiatric symptoms can be heralded by intense headache followed by any combination of altered behaviour, agitation, aggression, suicidal ideation, confusion and delusions.^145^ Historically, such features could have been accepted to occur as a natural consequence of cerebral damage incurred directly from the virus. In this scenario, testing for paired serum and CSF autoantibodies is necessary—especially NMDAR IgG antibodies—with CSF antibodies more often identified. This may reflect testing using commercial rather than live cell-based assays; with positivity from the latter demonstrating greater clinical significance in primary AE.^146^ Other antibodies that have been found in association include the those targeting the dopamine 2 receptor, CASPR2, LGI1, GABA_A,_ GFAP and unknown neuronal cell surface receptor targets.^16^

Brain MRI changes are heterogenous in post-HSE AE, with more necrosis and cystic lesions versus non-relapsing HSE, in the areas of prior HSE damage.^16^ Markers of neuroglial damage, including neuron-specific enolase, GFAP and S-100B, are often within normal limits.^7^ This is in contrast to primary HSE, in which viral-induced cell lysis predominates.

A recent prospective cohort study involving 86 patients investigated potential HLA associations from patients with AE following HSE versus patients with HSE who did not develop autoimmune encephalitis, healthy controls and anti-NMDAR AE not related to HSE.^147^ The absence of the HLA-A*02 allele was deemed a risk factor for developing subsequent AE following HSE (4 of 21, 19%) versus HSE patients without autoimmune encephalitis (42 of 65, 65%) and healthy Spanish controls (2005 of 4335, 46.25%) but not in the control anti-NMDAR AE group (21 of 36, 58.33%). HLA-A*02 single nucleotide polymorphisms have been associated with a decreased B and T cell response to type I IFNs.^148^ Indeed, the authors suggest this, together with altered viral clearance or negative thymic selection, may indirectly be involved in the predisposition to AE post-HSE.^147^ Further independent population-based studies will be essential to replicate the association with post-HSE AE.

In the case of HSE, autoantibodies are generated in almost one-third of patients; with the majority targeting the NMDA receptor and predominantly IgG1.^149,150^ NMDAR-specific IgA and IgM antibodies have also been identified in the CSF,^134^ and chemokines such as CXCL13, CCL19 and a proliferation-inducing ligand (APRIL) were elevated in post-HSE anti-NMDAR AE.^45^ This phenomenon of immunoglobulin class-switching and chemokine production provides evidence to support the role of germinal centre activity in secondary autoimmunization post-HSE (Box 2).

Box 2 **Germinal centre reactions**

Germinal centres are specialized niches within secondary lymphoid tissue, which produce memory B cells and long-lived plasma cells. Fastidious germinal centre refinement of the B-cell receptor pool for a specific antigen generates clonal expansion of selected B cells, honing the immune response.

Upon antigen presentation—either directly, via dendritic cells (follicular and non-follicular) or macrophages—B cells subsequently upregulate their C-C motif chemokine receptor 7 (CCR7) to facilitate migration to a nearby T cell zone in which CCR7 ligands (CCL19 and 21) are abundantly expressed.^151^ B cells subsequently present antigen fragments on MHC-II to CD4^+^ T-helper cells. This B–T cell interaction determines the survival and co-stimulation of B cells, which can then initiate a germinal centre response.

Selected B cells can now undergo migration into germinal centres where they have the opportunity to alter their respective receptor antigenic binding properties through somatic hypermutation (SH). SH is initiated by the activation-induced cytidine deaminase (AID) enzyme, which binds to single stranded DNA to deaminate cytosine (C) into the highly mutagenic deoxy-uracil (U); increasing the mutation rate in the immunoglobulin genes by 1 000 000-fold.^152^ These genetic iterations allow for a broad antibody repertoire, which consists of at least 1011 different specificities.^153^

B cells subsequently upregulate their expression of CXCR5 facilitating migration towards the light zone via its respective chemoattractant—chemokine CXCL13. CXCL13 is released from follicular dendritic cells and, to a lesser extent, T follicular helper (TFH) cells when activated by an antigen presenting follicular dendritic cell.^154,155^ B cells now have the opportunity to undergo class-switch recombination, which is also facilitated by AID. AID creates double-stranded DNA breaks followed by recombination in the constant region of the immunoglobulin heavy chain.

### Biological mechanisms

The concept of immune privilege in the CNS was first proposed in the late 1940s by Medawar who found that skin homografts transplanted to the brain failed to elicit an immune reaction.^156^ This concept may be relevant in post-HSE AE, supporting the hypothesis that CNS damage leads to exposure of CNS-privileged epitopes into the periphery generating targeted neuroinflammation. Alternative plausible hypotheses include non-specific B-cell activation and molecular mimicry. The latter has been supported by the observation that patients with classical anti-NMDAR AE, not related to HSE, had higher rates of HSV antibodies versus age-matched controls.^157^ However, in view of the broad array of autoantibodies with known and unknown antigenic targets, together with reports of non-HSV viral triggers release of CNS epitopes to the periphery is regarded as most likely.

The mechanism by which neuroglial surface proteins drain outside of the CNS has been long questioned. Traditionally accepted mechanisms of CSF drainage include three processes: drainage through arachnoid granulations,^158^ crosstalk between a poorly defined route located along the olfactory nerves and the nasal lymphatics via the cribriform plate^159^ [which then drain to the cervical lymph nodes (CLNs)] and via apical cell transporters along the choroid epithelium. Although these mechanisms remain important, recent studies have challenged the neuroimmunological dogma and provide evidence supporting the meningeal lymphatics as the dominant drainage system from the brain to the periphery.^118-121^ The *bona fide* lymphatics within the CNS meninges are a network of thin-walled structures with fewer ramifications in comparison to their peripheral lymphatic counterparts.^118^ These exit the cranium and converge along the sigmoid sinus, retroglenoid vein and meningeal portion of the pterygopalentine artery.^118-120^ The meningeal lymphatic function is to drain small molecules and immune cells from the subarachnoid space and CNS fluid from the CSF and interstitium to the dCLNs—and to a lesser extent, the superficial CLNs.^119,120,160,161^ As demonstrated in other neurological disorders (Box 3), neuroglial host antigenic exposure within the periphery occurs in response to CNS damage. Importantly, many of these neuroglial CNS proteins possess the potential to bypass immune tolerance mechanisms within germinal centres with subsequent peripheral clonal expansion and development of NSAb-mediated diseases.^162^ More specifically, further theories for secondary AE include co-presentation of viral PAMPs and cerebral ‘neo-antigens’ to NMDAR antibody-specific B cells in draining peripheral lymph nodes, which trigger TLR B-cell signalling facilitating tolerance escape.^163,164^

Box 3 **Neuroglial surface antibodies in other neurological disorders**

Neuroglial surface antibodies following neuronal damage have been identified in other neurological conditions, which damage the structural integrity of the brain, including traumatic brain injury (TBI), ischaemic stroke and Alzheimer's disease.^165-167^

In TBI, a microarray study identified the presence of autoantibodies, predominantly IgM, targeting a wide variety of antigens.^165^ These autoantibodies developed in the first week following TBI and often remained for many years. The clinical and immunological relevance of these polyreactive autoantibodies is currently unknown.

Brain-derived antigens have also been identified within palatine tonsil and cervical lymph node tissue in acute stroke patients.^168^ Indeed, myelin basic protein, NR2A subunit of the NMDAR and myelin oligodendrocyte glycoprotein (MOG) were identified. These antigens were found in the presence of brain antigen immunoreactive antigen-presenting cells. Notwithstanding, only a minority [2/60 (3.3%) at 90 days] of patients develop autoantibodies against neuroglial antigens.^169^

Finally, in Alzheimer’s disease, which is also associated with damage to the brain's structural integrity, a longitudinal increase in CSF B cell numbers in association with cerebral amyloid-β (Aβ) deposition has been identified.^167^ B cell receptor (BCR) profiling identified commonalities between different Alzheimer’s disease patients, who exhibited similar class-switched BCR sequences, was absent in control patients. Interestingly, levels of antibodies targeting different epitopes of Aβ were associated with the rate of cognitive decline in patients with Alzheimer’s disease. Moreover, B cell depletion was associated with reduction of Aβ deposition, restoration of microglial transforming growth factor β production and the arrest of cognitive decline in Alzheimer’s disease mouse models.^170^

Overall, it may be that the common theme of damage to the structural integrity of the brain is not enough for privileged neuroglial epitopes to facilitate escape from peripheral tolerance mechanisms alone and generate CNS autoantibodies. It is conceivable that antigen release plus the degree of the disease-specific immune response is important—together with other predisposing genetic factors such as patient HLA status—however, further evidence is required.

**Figure 2:**
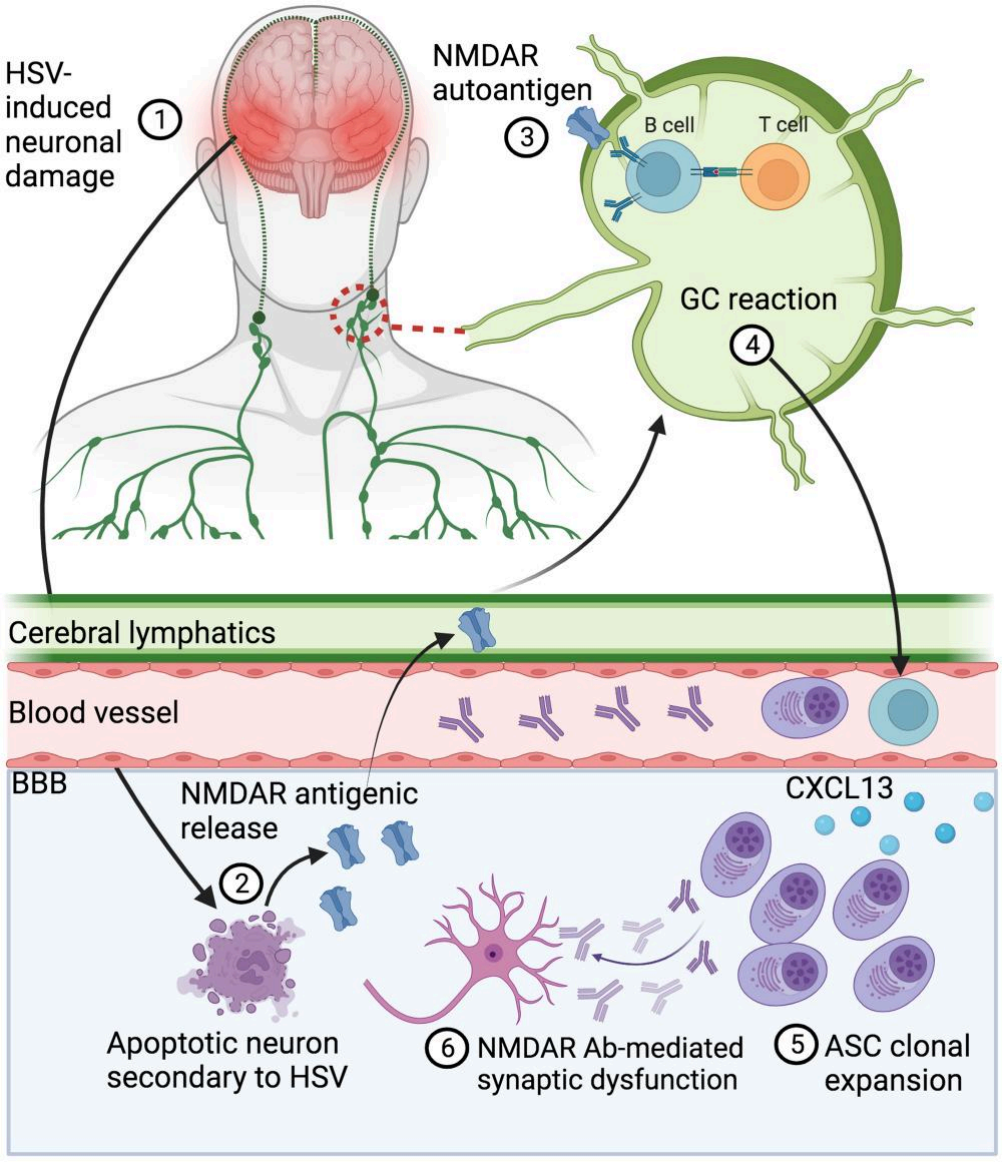
**Proposed mechanism of secondary autoimmunization post-HSE.** The deep cervical lymph nodes are implicated here as the primary peripheral lymphatic drainage site from the meningeal lymphatics of the CNS with a schematic overview of germinal cell (GC) reactions and subsequent inflammatory milieu leading to CNS antibody-mediated neuronal dysfunction (modified from Sun *et al*.^162^). Ab = antibody; ASC = antibody secreting cell; BBB = blood­–brain barrier, HSE = herpes simplex virus encephalitis; NMDAR = *N*-methyl D-aspartate receptor.

Two recent studies have implicated dCLNs in autoreactive B cell immunization.^154,171^ The exposure of B cells to CNS antigens results in a cascade of germinal centre reactions, including immunoglobulin affinity maturation, class switch recombination and diversification (Fig. 2). Indeed, class-switch recombination is a mechanism to shift the early antigen-specific IgM to the IgA or IgG isotypes and is of relevance to many autoantibody-mediated diseases including NMDAR-antibody encephalitis and aquaporin-4 neuromyelitis optica spectrum disorders.^154,171^

Finally, the biological rationale for the observed AE phenotypic dichotomy after HSE between young children versus older children and adults remains elusive. With increasing age, the innate immune system is primed towards a basal activation state. Alterations in DNA methylation and post-translational histone modifications increase inflammatory gene expression within T cells and monocytes.^172,173^ Ageing is also associated with increased neutrophils, activated CD14^+^CD16^+^ monocytes, natural killer cells and chronic low-level secretion of pro-inflammatory cytokines.^174-176^ In children, the immunobiology for post-HSE AE is even less clear. According to our data, children ≤4 years who develop post-HSE AE have poorer outcomes. However, it is unclear whether the observed outcomes are resultant from the severity of the index HSE and/or the subsequent brain-directed autoimmunity. Overall, early host antiviral mechanisms, immune tolerance, chronic inflammation and immunosenescence vary throughout life and may all influence the risk-profile for developing post-HSE AE. This expansive topic has been reviewed extensively elsewhere.^172,177,178^

### Treatment of post-herpes simplex virus encephalitis autoimmune encephalitis

Although not fully substantiated, first-line therapy for post-HSE NMDAR-antibody AE parallels traditional NMDAR-antibody AE^140^ in the form of high-dose corticosteroids with or without plasma exchange (PLEX) or intravenous immunoglobulins (IVIg). Second-line therapy includes rituximab or cyclophosphamide. The benefit of medium-term immunomodulation is unknown. In a prospective cohort study of 51 patients with post-HSE AE, patients with persistent autoantibodies at 1-year follow-up were more disabled (median mRS 3 versus 2, *P* < 0.001) and more frequently prescribed anti-seizure medications (71% versus 35%, *P* = 0.046).^16^ Older children and adult patients receiving first-line treatment typically demonstrate a favourable outcome assessed at 1-year follow-up with reduction in NMDAR antibody titres.^16,145,179-181^

## Autoimmune encephalitis following other neuroinfectious diseases

Although HSE is the most widely reported neuroinfectious disease associated with subsequent AE, the literature has expanded to include other possible infective precipitants including varicella zoster virus,^182-184^ Japanese encephalitis (JE),^185,186^ Epstein-Barr virus,^187^ chikungunya,^187^ tick-borne encephalitis,^188^ HIV,^189^ human herpesvirus 6 (HHV-6)^187^ and 7 (HHV-7),^187^ tuberculous meningitis^190^ and an unusual case suggestive of preceding *Angiostrongylus cantonensis* infection.^191^ Intriguingly, and mirroring AE following HSE, most of these studies report detection of autoantibodies against the NMDAR and predominantly within the CSF, which are generally deemed more clinically relevant than their serum counterparts when analysed through fixed cell assays.^192,193^

Intriguingly, pro-inflammatory cytokine signatures similar to HSE have been observed in patients with Japanese encephalitis. An observational study of 118 Vietnamese patients infected with the JEV found that CSF IL-6, IL-8 and IFN- α elevation were associated with poorer outcomes.^194^ In contrast, survivors had higher levels of IgM and IgG within the CSF and IgM in plasma. Of note, clinical correlations in these patients between elevated immune markers and longitudinal progression to AE were not reviewed.

The rationale for the emergence of NMDAR AE after different neuroinfectious diseases is unknown. The observation that it follows neurotropic infections that span multiple pathogens suggests broad pathological mechanisms in need of further research.

## Future perspectives: from disease biology to immunotherapy

**Figure 3:**
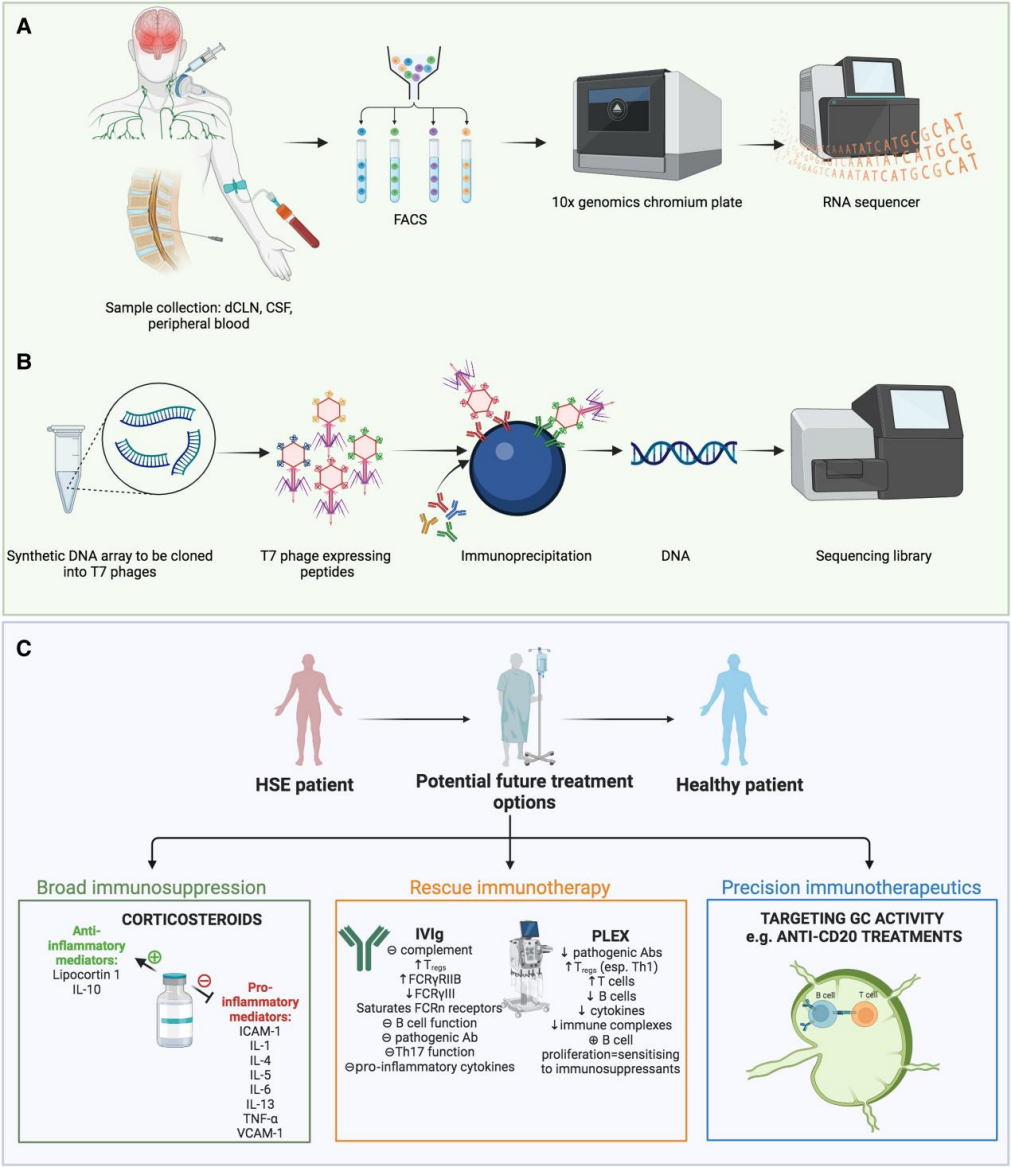
**Future perspectives in HSE research and possible immunotherapeutic options.** (**A**) Sampling from different compartments in the body from patients with herpes simplex virus encephalitis (HSE), including peripheral blood, CSF and deep cervical lymph nodes (dCLNs) is illustrated. Immune cells can be separated using fluorescence-activated cell sorting (FACS) and individual populations can be analysed using high-throughput single cell RNA-sequencing through a 10× genomics chromium plate—for example—and analysed using an RNA sequencer to generate intercompartmental genomic expression data. (**B**) Phage-immunoprecipitation and sequencing (PhIP-Seq) process begins with a T7-peptide library generated from DNA sequences encoding 36-amino acid peptides from 24 329 open reading frames. DNA sequences are amplified and cloned into a T7 phage, which is then mixed with patient samples containing autoantibodies for immunoprecipitation by capture via magnetic beads. Immunoprecipitated DNA from respective phages are recovered and PCR amplified to be stored and analysed within a sequencing library. (**C**) Illustrates potential immunotherapeutic options in patients with HSE or post-HSE autoimmune encephalitis that require further research to support their clinical utility. ⊕ = stimulation; ⊖ = inhibition; FcγR = the receptors for the Fc region of IgG; ICAM-1 = intercellular cell adhesion molecule 1; IVIg = intravenous immunoglobulin; PLEX = plasma exchange; Th = T helper cells; Treg = regulatory T cell; VCAM-1 = vascular cell adhesion molecule 1.

Insights into the adaptive immunobiology of HSE and post-HSE AE, at the cellular level, can be explored with advances in the field of single cell profiling, using high-throughput B-cell receptor (BCR) and T-cell receptor (TCR) sequencing (Fig. 3A). Given the massive enrichment of cells that undergo clonal expansion, a well powered study can be performed using limited patient numbers—an important concept in rare diseases. Following sequencing, BCRs or TCRs can be cloned into eukaryotic expression vectors for functional analysis. These can then be tested for reactivity against an array of self- and non-self-antigens. Indeed, relevant to neuroinflammation, these techniques have been recently explored in patients with multiple sclerosis demonstrating association with the Epstein-Barr virus (EBV),^195^ traditional anti-NMDAR AE^154^ and anti-aquaporin-4 neuromyelitis optica spectrum disorder (NMOSD).^171^ In the context of post-HSE AE, this methodology has potential to reveal NSAbs that are most pathogenic whilst assessing for overlapping affinity of clonally expanded BCRs or TCRs for HSV-1 and cerebral autoantigenic epitopes. Relevant immunobiological mechanisms can be inferred including the mutational distance from the germline, class-switch recombination and intercompartmental clonal overlap (i.e. between CSF, germinal centres and peripheral blood). Analysis of these processes can be harnessed for personalized therapeutic benefit—such as in the case for rituximab and NMOSD.^171^

Other major areas of proposed research providing further insights into the immunobiology of AE following HSE include functional assessment of patient-derived monclonal Abs (mAbs) in tissue and experimental models; recently coined ‘brain antibody-omics’.^196^ This could include binding analyses through immunohistochemistry on rodent or monkey brain sections and/or cell surface binding characteristics on live hippocampal neurons. Indeed, broad tissue-based analysis have recently been found helpful in identifying antigen-specific immunoreactive patterns from known NSAbs but whether mAbs derived from HSE patients exhibit different binding patterns remains to be determined.^197^ In addition, direct pathogenic effects could be observed at the cellular level through iPSC-derived neuroglial systems.^63^ Progressing further, stem cell-derived brain organoids have emerged as a model of human brain development, physiology and tissue architecture.^198,199^ In recent times, brain organoids have also been used to study HSE and other neurotropic viral diseases.^200,201^ Results derived from early work analysing direct consequences from infection with HSV-1 identified massive reductions in neuronal activity with upregulation of TNF signalling prevented through combinatorial antiviral/anti-inflammatory treatment.^202^ Finally, informative animal models exploring passive intrathecal transfer of antibodies from post-HSE AE patients are important research avenues to explore synaptic transmission, behavioural changes and hippocampal NMDAR densities as previously described.^203,204^

Although the majority of cases of post-HSE AE are associated with autoantibody reactivity against known neuroglial antigens, just under one-quarter of patients develop secondary AE with autoimmunity targeting unknown antigens (Supplementary material). Mapping the epitopes in those patients with seronegative post-HSE AE is important to understand the immunopathogenesis of this disease process. Recently, a synthetic representation of the entire human proteome has been engineered for peptide display on the surface of a T7 phage.^205^ Patient serum can be used in phage-immunoprecipitation and sequencing (PhIP-Seq) to isolate and enrich specific T7 phages displaying autoantigens. Sequencing enriched T7 phage libraries can then be used to identify patient-specific epitopes (Fig. 3B). Indeed, PhIP-Seq has been successfully used to reveal autoantigens from neurological paraneoplastic syndromes associated with anti-Ma, anti-Ri, anti-Yo, anti-Hu^206^ and more recently anti-kelch-like protein 11 antibodies.^207^

The concept of harnessing properties of immunosuppression for clinical benefit in patients with HSE is an evolving area of research and particularly relevant given the devastating neuroinflammatory reaction patients typically incur. Indeed, a retrospective non-randomized logistic regression analysis of 45 HSE patients suggested corticosteroid administration, along with age and level of consciousness at initiation of aciclovir, was an important variable in determining a favourable outcome.^208^ There are multiple additional small case series/reports drawing similar conclusions in favour of corticosteroid treatment for acute HSE, although the treatment timing varies from simultaneous administration to several days following initiation of antiviral therapy.^209-213^ Corroborating experimental studies have also concluded that corticosteroid administration reduced MRI-brain abnormalities without increasing the brain parenchymal HSV-1 viral load.^214-216^ Moreover, corticosteroid treatment is associated with a significant decline in CSF IL-6 expression; a pro-inflammatory cytokine correlated with poorer outcomes in HSE.^42^

The German trial of aciclovir and corticosteroids in HSE (GACHE; ISRCTN45122933) was intended to address the role of immunosuppression in HSE but was inconclusive due to limited recruitment.^217^ Subsequently, the dexamethasone in HSE (DEX-ENCEPH; NCT03084783) trial, which is a multinational, randomized control trial (RCT), has recently completed recruitment and is due to report its preliminary findings soon, including the impact of steroids on the emergence of NMDAR autoantibodies at 26 weeks.

Our review of the literature has highlighted that almost one-half of patients with post-HSE AE are receiving second-line immunotherapy including rituximab or cyclophosphamide. One study longitudinally assessed the CSF and peripheral blood using flow cytometry throughout the treatment of rituximab.^218^ During maintenance treatment, activated T cells, B cells and plasma cells decreased in both compartments together with a decline in anti-NMDAR IgG and HSV IgG—without HSV reactivation. Despite previous conflicting human and animal data on the role of rituximab at depleting B-cell subsets from secondary lymphoid structures,^219-221^ recent evidence supports its role in selective attenuation of germinal centre activity in other neuroinflammatory diseases.^154,171^ In view of the likely role of germinal centres in secondary autoimmunization post-HSE, rituximab—or other anti-CD20 therapies—are highlighted as biologically plausible treatment options.

## Conclusion

HSE is a devastating disease with antiviral treatments being the core management strategy. Despite this, the morbidity and mortality rate remains high. The neuroimmunological evolution of HSE is an important research area. Further understanding could reveal genetic predispositions and novel treatment paradigms tailored to the particular stage of the disease. Moreover, the immunopathogenesis of post-HSE AE requires further study, with the aim to provide biological evidence to inform future clinical trials. Relevant peripheral biomarkers of germinal centre activation may stratify patients with HSE by their individualized risk of secondary autoimmunization. This could facilitate pre-emptive use of precision immunotherapeutics in those patients most likely to benefit from such treatments while simultaneously reducing exposure of low-risk patients to any potential risks associated with broad immunosuppression. Elucidation of the underlying biology at this CNS-peripheral neuroimmune interface is likely to translate directly to other research areas of neuroinflammation related to different forms of brain injury or viral-mediated neuroinflammation.

## Supplementary Material

awad419_Supplementary_Data
