# Supplementary material for: The immunobiology of herpes simplex virus encephalitis and post-viral autoimmunity
Source: Brain. 2023 Dec 13;147(4):1130–48. doi: 10.1093/brain/awad419 (PMC10994539; doi:10.1093/brain/awad419)
Supplement: awad419_Supplementary_Data [file awad419_supplementary_data.pdf]

Literature review

To review the phenotype, management and outcome of post-HSE autoimmune encephalitis (AE) patients, a comprehensive literature search of Medline and Embase (Ovid) was undertaken from 2007 – when anti-NMDAR encephalitis was originally discovered[1] – to March 2023 using text words and subject headings relating to "herpes simplex" AND "encephalitis" AND "autoimmune encephalitis". A total of 627 publications were screened and abstracts were reviewed. Publications excluded in the analysis included: conference proceedings, non-full text publications, non-English translated manuscripts, reports without individual patient data for analysis, patients not fulfilling the diagnostic criteria for encephalitis[2] and those without originally confirmed HSV DNA within the CSF identified by polymerase chain reaction (PCR). A total of 32 publications were deemed suitable for full analysis, which included a total of 110 patients with summary characteristics highlighted (Supplementary Table 1). Studies that were included and had missing patient data for individually stratified characteristics were excluded from that particular comparison and detailed in the figure legend.

Statistical analysis

Analyses were performed in Excel (Microsoft) and Prism (Graphpad). Proportions were compared using the Chi squared test. Analysis included clinical features, investigatory findings, management and outcomes of patients with post-HSE AE. Patients with secondary AE were divided by age (≤4 and >4 years) based on previous work highlighting a dichotomy in phenotypes and outcomes[3–5]; especially in comparison to younger children[6]. All variables with a cut off p value of less than 0.05 were considered significant.

Supplementary Table 1 Summary characteristics of post-HSE AE patients.

**Demographics and overview**			
Total number of cases	110		
Number ≤ 4 years	44		
Number > 4 years	66		
Median age of onset, range	13.5 (2 months- 84 years)		
Female:Male	1.4:1		
Median number of days from HSE symptoms onset to secondary AE, range	30 (7-510)		
**HSE clinical features (n=59)[a]**			
Fever	50 (84.8%)		
Encephalopathy	49 (83.1%)		
Seizures	38 (64%)		
Focal neurological deficit	16 (27.1%)		
**Post-HSE AE clinical features**	**≤4 years**	**>4 years**	**Comparison**
Behavioural change[b]	38 (86.4%)	51 (77.3%)	p=0.2346
New seizures	21 (47.7%)	22 (33.3%)	p=0.1296
Movement disorders (choreoathetosis, dystonia, dyskinesias, automatisms)	43 (97.7%)	18 (27.3%)	p=<0.0001****
Encephalopathy	33 (75%)	29 (43.9%)	p=0.0013**
Autonomic dysfunction	9 (20.5%)	14 (21.2%)	p=0.9237
Insomnia	16 (36.4%)	11 (16.7%)	p=0.0187*
New focal neurological deficit	3 (6.8%)	11 (16.7%)	p=0.1289
**Antibodies (n=111)[c]**	**≤4 years (n=44)**	**>4 years (n=67)**	**Comparison**
NMDA receptor	39 (90.7%)	42 (62.7%)	P=0.0026**
LGI1 receptor	0 (0%)	1b (1.5%)	p=0.4156
CASPR2 receptor	0 (0%)	1 (1.5%)	p=0.4121
$GABA_A$ receptor	1a (2.3%)	0 (0%)	p=0.2151
DA2R receptor	1 (2.3%)	0 (0%)	p=0.2151
GFAP receptor	0 (0%)	2 (3%)	p=0.2475
Unknown antigens	3 (7%)	21 (31.3%)	p=0.0021**
**Radiological features (n=42)**	**≤4 years (n=13)**	**>4 years (n=29)**	**Comparison**
MRI-brain abnormalities at HSE presentation	13 (100%)	28 (96.6%)	p=0.498

New or progressive MRI-brain changes at AE relapse	3 (23.1%)	14 (48.3%)	p=0.124
**Immunotherapy[d]**	**≤4 years (n=44)**	**>4 years (n=66)**	**Comparison**
No immunotherapy	2 (4.5%)	8 (12.1%)	p=0.1757
First line	42 (95.4%)	58 (87.9%)	p=0.1757
Second line	23 (52.3%)	23 (34.8%)	p=0.0695
**Outcome**	**≤4 years (n=43)**	**>4 years (n=56)**	**Comparison**
Full recovery (n=99)	1 (2.3%)	5 (8.9%)	p=0.0468*
Good outcome (mRS ≤2) (n=99)	4 (9.3%)	22 (39.3%)	p=0.0010***
Poor outcome (mRS >2) (n=99)	38 (88.4%)	29 (51.8%)	p=<0.0001****

[a]Cohort B from Armangue et al 2018[3]and Brás A et al 2020[7] (n=50) were excluded from HSE clinical features given the lack of descriptive data. Further studies were excluded from the radiological features section and outcome due to paucity of information. [b]Behavioural features in the very young (≤4 years) includes irritability and poor response to stimuli.

[c]Overlapping NSAbs from a single patient included.

[d]First line immunotherapy includes steroids and/or PLEX and/or IVIg. Second line immunotherapy includes RTX and/or CYC. ** p-value for difference between proportions.

*P<0.05, **P<0.01, ***P<0.001, ****P<0.0001. CASPR2 = contactin-associated protein-like 2; CYC = cyclophosphamide; D2R = dopamine 2 receptor; $GABA_A$ = γ-Aminobutyric acid type A; GFAP = glial fibrillary acidic protein; IVIg = intravenous immunoglobulin; LGI1 = leucine-rich glioma inactivated 1; NMDA = N-methyl-D-aspartate; PLEX = plasma exchange; RTX = rituximab.

Supplementary Table 2 Demographics, clinical features and outcomes of post-HSE AE patients acquired from the literature search

Reference	*Number of patients*	*Age (years)*	*Gender*	*Time (days) from HSE onset to AE*	*Clinical features*	*NSAb*	*Outcome*
Ayvacioğlu Çağan C et al. Life After Tetra Hit: Anti-NMDAR Encephalitis After HSV Encephalitis in a NMOSD Coexistent with Sjögren's Syndrome. Noro Psikiyatr Ars. 2022 Apr 12;59(2):161-163.	*1*	*52*	*F*	*26*	***HSE**: altered mental status with memory impairment and expressive dysphasia despite aciclovir **AE**: persistent altered mental status with memory impairment and expressive dysphasia despite aciclovir*	*NMDA (CSF only)*	*able to walk and cooperate in time, space and person.*
Berek K, et al. Caspr2 antibodies in herpes simplex encephalitis: an extension of the spectrum of virus induced autoimmunity? - A case report. BMC Neurol. 2022 Apr 5;22(1):131.	*1*	*82*	*F*	*13*	***HSE**: aphasia with leading deficits in verbal fluency and memory performance **AE**: stable clinical symptoms*	*CASPR2 (serum only)*	*mRS 3 at 7 months follow-up*
Gomez et al 2021. The firs recorded case of herpes simplex virus encephalitis followed by anti-NMDA receptor autoimmune encephalitis after resection of meningioma. J Interdisciplinary Neurosurgery; 24:1-3.	*1*	*24*	*M*	*44*	***HSE**: 10 days post-operative exhibited fever, headache, olfactory hallucinations **AE**: psychotic behaviour, trichotillomania, aggressiveness 44 days post HSE symptom onset*	*NMDA (compartment not discussed)*	*Clinically stable at 6 months without new neurological sequelae*
Hu S et al. HSV encephalitis triggered anti-NMDAR encephalitis: a case report. Neurol Sci. 2021 Mar;42(3):857-861.	*1*	*23*	*M*	*LP performed on d11 post-symptom onset*	***HSE**: Seizures, mood instability, irritability, pathological laughter and*	*NMDA (serum and CSF), GAD (serum only)*	*Symptoms were well controlled with*

				detecting NMDAr antibodies	*crying, fever, disorientated, hypotonia.*<br><br>***AE**: concomitant presentation*		*only mild cognitive deficits at the 1-year follow-up (mRS = 1)*
Jiang Y et al. Virus reactivation after immunotherapy of anti-NMDAR encephalitis secondary to herpes simplex encephalitis: A case report. Brain Dev. 2021 Nov;43(10):1057-1060.	*1*	*2.5*	*F*	*32*	***HSE**: Fever, seizure, left limbs weakness and left-sided central facial palsy*<br><br>***AE**: aphasia and seizure (right limb tonic without impaired consciousness), disturbance of consciousness, decreased amount of sleep and involuntary movement of limbs and mouth. Recurrence of HSV occurred on day 58 following rituximab.*	*NMDA (CSF)*	*Discharged with seizure-free, less involuntary movement and improvement of consciousness disorder.*
Manglani M et al. Anti-NMDAR Encephalitis After Neonatal HSV-1 Infection in a Child With Low TLR-3 Function. Pediatrics. 2021 Sep;148(3):e2020035824.	*1*	*2*	*M*	*22*	***HSE**: Fever, altered mental status, and seizures*<br><br>***AE**: emotional outbursts, focal seizures, and milestone regression including speech loss and reversion to crawling; emotional outbursts, focal seizures, and milestone regression including speech loss and reversion to crawling*	*NMDA (CSF)*	*After treatment, the patient had elevated anti-NMDAR antibodies and neurologic deficits including a limited ability to talk with an ~30-word vocabulary, irregular sleep, staring spells, and behavioral outbursts. At 6 years old, he is still receiving daily acyclovir and monthly IVIG. He did not develop other recurrent or unusual infections during this period. His most recent neuropsychological evaluation revealed an improvement in cognitive, gross, and fine motor skills. His vocabulary is expanding and he is able to vocalize his needs with 2- to 3-word sentences. He enjoys simple puzzles and playing with his grandmother.*

Swayne A et al. Analysing triggers for anti-NMDA-receptor encephalitis including herpes simplex virus encephalitis and ovarian teratoma: results from the Queensland Autoimmune Encephalitis cohort. Intern Med J. 2022 Nov;52(11):1943-1949.	*3*	*43-68 (mean 57)*	*M:F 1:2*	*22-100*	***HSE**: rapid encephalopathy (100%) + systemic signs of infection* ***AE**: 2/3 viral prodrome, cognitive impairment (100%), mood disorder (2/3), seizures (2/3), status epilepticus (1/3), weakness (2/3), autonomic dysfunction (2/3), ataxia (1/3), movement disorder (1/3).*	*NMDA (CSF)*	*mRS = 1.67 (average final)*
Brás A et al. Anti-NMDAR Encephalitis Following Herpes Simplex Virus Encephalitis: 2 Cases From Portugal. Neurohospitalist. 2020 Apr;10(2):133-138.	*2*	*50 and 33*	*F*	*~28*	***HSE**: "acute encephalitis"* <br>***AE**: 1) orofacial and right foot dyskinesias, dysautonomia, and focal seizures with poor response to levetiracetam* *2) Week 4, mild personality change which progressed rapidly to hypoactive delirium, audio-visual hallucinations, severe short-term memory, language, executive–visual–spatial functions impairment, left limb apraxia, and pyramidal signs.*	*NMDA (CSF and serum in patient 2)*	*1) mRS = 2 2) mRS = 0*
Peters J, Wesley SF. Case of concurrent herpes simplex and autoimmune encephalitis. Neurol Neuroimmunol Neuroinflamm. 2020 Oct 2;7(6):e897.	*1*	*84*	*F*	*21*	***HSE**: 3 days of progressive dizziness, fevers, and confusion.* <br>***AE**: 1 week following aciclovir developed poor attention and followed only simple commands. She had intermittent episodes of brief left facial grimacing with contraction of the left arm consistent with faciobrachial dystonic seizures. Serum sodium 123.*	*LGI1 (serum, 1:20 and CSF) NMDA +ve CSF (1:2 negative)*	*Hospice care 4 months post-discharge*

Armangue T et al. Toll-like receptor 3 deficiency in autoimmune encephalitis post-herpes simplex encephalitis. Neurol Neuroimmunol Neuroinflamm. 2019 Sep 5;6(6):e611.	*1*	*6*	*F*	*5 months*	***HSE**: acute-onset headache, fever, decreased level of consciousness, aphasia, and right hemiparesis*<br><br>***AE**: Five months later, she was readmitted with severe headache and decreased level of consciousness*	*Unknown antigen (CSF immunochemistry studies on rat brain tissue and cultured live neurons showed strong reactivity revealing the presence of neuronal antibodies)*	*"neurological improvement"*
Handoko M et al. Autoimmune Glial Fibrillary Acidic Protein Astrocytopathy Following Herpes Simplex Virus Encephalitis in a Pediatric Patient. Pediatr Neurol. 2019 Sep;98:85-86.	*1*	*12*	*M*	*2 months*	***HSE**: headache, fever, vomiting, decreased responsiveness, and seizures*<br><br>***AE**: increasing memory deficits, impulsivity, and behaviour problems. During this time, he was also diagnosed with migraines and started on topiramate. Around one year after his initial HSE, his symptoms acutely worsened and included disinhibition, impulsivity, hypersexuality, hypersomnia, hallucinations, aggression, and eventually suicidal ideation for which he was briefly admitted to an inpatient psychiatric facility.*	*GFAP (serum and CSF)*	*Waxing and waning neuropsychiatric symptoms. He has returned to school, but continues to struggle with cognitive defi- cits*
Yan Hung SK, Hiew FL, Viswanathan S. Anti-NMDAR Encephalitis in Association with Herpes Simplex Virus and Viral and Bacterial Zoonoses. Ann Indian Acad Neurol. 2019 Jan-Mar;22(1):102-103.	*1*	*44*	*M*	*14*	***HSE/AE combined**: Progressive language difficulty and intermittent right facial and upper limb twitching for 2 weeks, followed by aggressive behaviour. There was marked orofacial dyskinesias and faciobrachial dystonia. A series of right head jerky aversive seizures were observed, with progression to involve ipsilateral limb and*	*NMDA (serum and CSF)*	*Residual mild receptive dysphasia and cognitive deficits (Mini-Mental State Examination 24/30).*

					subsequently prolonged generalized seizures requiring invasive mechanical ventilation.		
Ko JM et al. Hyperammonemia in a case of herpes simplex and anti-N-methyl-d-aspartate receptor encephalitis. Brain Dev. 2019 Aug;41(7):634-637.	*1*	*20 months*	*F*	*21*	***HSE**: She developed a high fever with-<br>out any prodromal symptoms. The day after fever onset,<br>a left-sided tonic–clonic seizure lasted for 20 min, and<br>her mental status became drowsy.<br><br>**AE**: Three weeks after<br>initial fever onset, fever developed again. She became<br>extremely irritable, and her mental status deteriorated.<br>She also showed orofacial dyskinesia and choreiform limb<br>movement.*	*NMDA (serum and CSF; 1:1280)*	*During 10 months of treatment and rehabilitation, her neurologic status gradually<br>improved. She became alert and reacted responsively.<br>However, she could not communicate verbally. She also<br>had difficulty swallowing. She could sit alone and stand<br>with assistance. Irritability and dyskinesia were partially<br>improved but persisted. There were no definite abnormalities on neurologic examination except truncal and<br>extremity hypotonia*
Mrad L et al. Severe presentation of antibody-negative, postinfectious steroid-responsive encephalitis and atonic bladder after herpes simplex encephalitis. BMJ Case Rep. 2019 Jul 22;12(7):e230005.	*1*	*75*	*F*	*28*	***HSE:** 3 days confusion, moderate headache, intense episodic dizziness and visual hallucinations.<br><br>**AE**: Four weeks from initial presentation, after finishing the acyclovir course, the patient was readmitted with worsening encephalopathy and the development of new generalised choreiform movements, worse in right arm.*	*Unknown antigen (no live neurone-based assay)*	*Three months post discharge, she showed progressive improvement at follow-up. A Montreal Cognitive Assessment Test Score improved to 26/30 compared with 14/30 in the rehabilitation unit.*

Sahar N, Nurre AM, Simon RQ. Infectious Trigger for Autoimmune Encephalitis: A Case Report and Literature Review. Case Rep Infect Dis. 2019 Nov 6;2019:5731969.	*1*	*61*	*M*	*8 months*	***HSE**: confusion and somnolence. On arrival, he was hemodynamically stable, and physical examination was significant only for delayed recall and poor concentration.*<br><br>***AE**: 8 months later agitation, behavioural changes, and confusion*	*NMDA serum (1:80) CSF (1:64 then 1:10)*	*He continued to suffer from intermittent episodes of combativeness, despite being on antipsychotic medications lacosamide and divalproex. He also experiences night-time awakenings with confusion and has not achieved his baseline personality or cognitive function.*
Alexopoulos H, et al. Post-herpes simplex encephalitis: a case series of viral-triggered autoimmunity, synaptic autoantibodies and response to therapy. Ther Adv Neurol Disord. 2018 Apr 23;11:1756286418768778.	*3/5 (2 excluded)*	*9 months, 14y, and 58y*	*All females*	*1) 23*<br>*2) 1 year*<br>*3) 6 months*	***HSE**: 1) Fever, staring;*<br>*2) fever, headache, confusion, seizures;*<br>*3) fever, headache, drowsiness*<br>***AE**: 1)choreoathetotic/dystonic movements and seizures; 2) complex partial seizures, limb jerks, somnolent, apnoeas, bradycardia (autonomic);*<br>*3) prosopagnosia, personality changes, generalised seizures*	*1) NMDA (serum and CSF)*<br>*2) NMDA (serum but -ve CSF),*<br>*3) NMDA (serum and CSF)*	*1) significant neurodevelopmental delay, mainly in speech, cognitive and social skills. Her motor development is better, without abnormal muscle tone, and a steady but slow improvement in her gross motor skills.*<br>*2) "clinically improved"*<br>*3) "clinically stable"*
Armangue T et al. Spanish Herpes Simplex Encephalitis Study Group. Frequency, symptoms, risk factors, and outcomes of autoimmune encephalitis after herpes simplex encephalitis: a prospective observational study and retrospective analysis. Lancet Neurol. 2018 Sep;17(9):760-772.	*62*	*Cohort A: 2/12 – 80y Cohort B: 4 months – 81y*	*M:F 1.75:1 (8/14 M)*	*Median 26 (<4yo), 43 (>=4yo)*	***HSE**: no full details*<br><br>***AE**: Change behaviour (54/58), seizures (22/58), choreoathetosis (27 - all <4), reduced LoC (33), dysautonomia (15)*	*NMDA 42, 1 GABAA, (others - unknown antibodies)*	*Median mRS 4 (<4yo), 2 (>=4yo)*
Li J et al. Autoimmune GFAP astrocytopathy after viral encephalitis: A case report. Mult Scler Relat Disord. 2018 Apr;21:84-87.	*1*	*35*	*F*	*5 months*	***HSE**: subacute headache, vomiting, fever.*<br>***AE**: including seizure and psychiatric/behavioral abnormalities*	*GFAP (serum and CSF)*	*After 1 month of treatment, headache, fever, and seizure resolved, MMSE and MoCA*

							scores increased (25/30 and 21/30, respectively),
Omae T et al. Cytokine/chemokine elevation during the transition phase from HSV encephalitis to autoimmune anti-NMDA receptor encephalitis. Brain Dev. 2018 Apr;40(4):361-365.	*1*	*3*	*F*	*28*	***HSE**: fever, headache, seizures*<br><br>***AE**: progressive irritability, movement disorder (chorea, dystonia, and stereotypic movements), and autonomic disturbances, such as tachycardia, low-grade fever, and insomnia*	*NMDA (CSF)*	*mRS = 0*
Kothur K et al. Cerebrospinal fluid cyto- /chemokine profile during acute herpes simplex virus induced anti-N-methyl-d-aspartate receptor encephalitis and in chronic neurological sequelae. Dev Med Child Neurol. 2017 Aug;59(8):806-814.	*1*	*13 months*	*F*	*31*	***HSE**: fever for 5 days, progressive lethargy, and left focal seizures with left-sided weakness*<br><br>***AE**: progressive irritability, autonomic disturbances in the form of tachycardia, low-grade fever, and insomnia. On day 31, she developed severe dyskinesias in the form of tongue thrusting, loud vocalization, and fast, continuous, fairly symmetrical large-amplitude choreoathetoid movements of her limbs, trunk and neck*	*NMDA (CSF)*	*Good progress in development, except for mild speech delay*
Nosadini M et al. Herpes simplex virus-induced anti-N-methyl-d-aspartate receptor encephalitis: a systematic literature review	*1*	*9 months*	*M*	*24*	***HSE**: fever, partial seizures, and encephalopathy*	*NMDA (CSF)*	*He now attempts to roll over, recognizes parents and responds by nonverbal cues, and gets occasional partial seizures.*

with analysis of 43 cases. Dev Med Child Neurol. 2017 Aug;59(8):796-805.					***AE**: new-onset orofacial dyskinesias and choreoathetoid movements of limbs along with recurrence of fever and seizures*		
Strippel C, et al. Treating refractory post-herpetic anti-N-methyl-d-aspartate receptor encephalitis with rituximab. Oxf Med Case Reports. 2017 Jul 3;2017(7):omx034.	*1*	*67*	*F*	*3 months*	***HSE**: aphasia and reduced consciousness*<br><br>***AE**: hallucinating, disorientated, aphasic and unable to verbally or non-verbally communicate. Muscle tone was increased with generalized rigidity, rendering the patient non-ambulatory.*	*NMDA (serum, 1:320 and CSF, 1:1000)*	*She was able to walk without support and to complete the activities of daily living nearly independently. At the 15 months follow-up whilst neuropsychological assessment performance had improved in all tests, certain residual deficits remained*
Geoghegan S et al. Anti-N-Methyl-D-Aspartate Receptor Antibody Mediated Neurologic Relapse Post Herpes Simplex Encephalitis: A Case Series. Pediatr Infect Dis J. 2016 Aug;35(8):e258-61.	*3*	*1) 15 months 2) 5 months 3) 16 months*	*1) F 2) M 3) F*	*1) 28 2) 27 3) 24*	***HSE**: 1) fever, focal seizures 2) poor feeding, fever, vomiting 3) lethargy, fever, focal seizure*<br><br>***AE**: 1) seizures and behavioural change, irritability, dramatic hemiballismus and choreiform movements*<br>*2) orofacial dyskinesia*<br>*3) altered consciousness, orofacial dyskinesia and chorea*	*NMDA (CSF)*	*1) She was discharged home 1 month later with dense right and mild left hemiplegia, language delay and bulbar difficulties requiring gastrostomy feeding. She remains hypogammaglobulinaemic and continues to receive IVIG replacement and valacyclovir*<br>*2) At 12 months of age, he had made good developmental progress without further seizures or abnormal movements.*<br>*3) At 6-month follow-up, she is ambulatory and the movement disorder has resolved*

Morris NA et al. HSV encephalitis-induced anti-NMDAR encephalitis in a 67-year-old woman: report of a case and review of the literature. J Neurovirol. 2016 Feb;22(1):33-7.	*1*	*67*	*F*	*3 months*	***HSE**: 3 days of confusion, personality changes, lethargy, headache, and fever*<br><br>***AE**: worsening confusion, disorientation, a mild occipital headache orofacial dyskinesias, neck pain, unintelligible mumbling and marked echolalia. She developed fixed nihilistic delusions as well as visual, auditory, and tactile hallucinations.*	*NMDA (serum and CSF)*	*10 months after initial HSV encephalitis onset, neurologic examination was normal, including formal neuropsychological testing. Her daughter reported only mild persistent personality changes*
Sutcu M et al. Role of Autoantibodies to N-Methyl-d-Aspartate (NMDA) Receptor in Relapsing Herpes Simplex Encephalitis: A Retrospective, One-Center Experience. J Child Neurol. 2016 Mar;31(3):345-50.	*2*	*1) 1yo 2) 3yo*	*F (both)*	*1) 21 2) 16*	***HSE**: 1) fever, seizure, encephalopathy 2) fever, encephalopathy, focal seizure*<br><br>***AE**: 1) refractory seizures, choreoathetotic and ballistic movements (head and trunk)<br>2) fever, irritability, choreoathetotic movement and dyskinesia*	*1) NMDA not available 2) NMDA (serum)*	*1) severe neurologic sequelae and multidrug therapy for hardly controllable seizures. Being 5 years old now, she has a pronounced developmental delay, intellectual disability, and ongoing epilepsy.<br>2) Currently, she is a 4-year-old girl who can walk and feed herself. Her seizures are under control with monotherapy. She has neurodevelopmental delay but is improving gradually with special educational therapy.*
Armangue T et al. Spanish Prospective Multicentric Study of Autoimmunity in Herpes Simplex Encephalitis. Autoimmune post-herpes simplex encephalitis of adults and teenagers. Neurology. 2015 Nov 17;85(20):1736-43.	*8*	*1) 13<br>2) 15<br>3) 45<br>4) 50<br>5) 34<br>6) 69<br>7) 29<br>8) 56*	*1) M<br>2) M<br>3) M<br>4) M<br>5) F<br>6) F<br>7) M<br>8) F*	*1) 42<br>2) 51<br>3) 44<br>4) 40<br>5) 38<br>6) 12<br>7) 21<br>8) 30*	***HSE**: 1) fever, headache and emesis. Over the next 2 days he developed aphasia, refractory seizures, and decreased level of consciousness<br>2) headache, focal seizures, and encephalopathy<br>3) sudden onset headache, fever, confusion, and speech problems*	*1) NMDA (CSF 1:160, serum 1:800),<br>2) NMDA (CSF, 1:80)<br>3) NMDA (CSF, 1:40; serum -ve),<br>4) NMDA (CSF, 1:2, serum -ve),*	*1) Partial improvement of behavioural deficits, F/U 15 mo, motor and cognitive deficits<br>2) rapid resolution of behaviour abnormalities, F/U 12 mo, complete recovery*

					4) subacute onset of fever, speech difficulties and memory deficits<br>*5) subacute onset of fever, speech difficulties, memory deficits, and focal seizures 6) speech difficulties and an acute confusional syndrome associated with fever*<br>*7) fever, seizures, and abnormal behaviour*<br>*8) low grade fever and diarrhoea followed the next day by apathy and somnolence*<br><br>***AE**: 1) 42 post-HSE he developed a drastic change in behaviour including prominent sexual disinhibition, cursing and insulting people, and aggressive behaviour, biting the pillow and objects. Also refractory HTN.*<br>*2) Fifty-two days after HSE onset he developed agitation, memory and cognitive deficits, and inappropriate behaviour including aggressiveness*<br>*3) acute onset headache, confusion, agitation, severe insomnia, and delusional thoughts*<br>*4) refractory headaches and behavioural symptoms 5) 38 days after HSE onset she developed progressive behavioural abnormalities, including anxiety, restlessness, delusional thoughts, irritability and insomnia*<br>*6) progressive increase in frequency of seizures that evolved to a non-convulsive status epilepticus*<br>*7) decreased level of consciousness and fever. At admission he was noted to have blepharospasm.*<br>*8) emotional lability, continuous crying, and suicidal ideation*	*5) NMDA (CSF, 1:40, serum 1:200) 6) unknown antigen (CSF and serum)*<br>*7) unknown antigen (CSF, serum not tested)*<br>*8) unknown antigen (CSF, absent in serum)*	*3) Relapsing symptoms that faded spontaneously, F/U 6 mo, residual aphasia*<br>*4) Improvement of behavioural symptoms, F/U 20 mo, moderate behavioral deficits*<br>*5) Improvement of behaviour, F/U 2 mo, mild aphasia and memory deficits*<br>*6) Transient response to PEX, seizure control post-RTX, F/U 3 mo, mild aphasia*<br>*7) Improvement after MP and local Botox, F/U 12 mo, minor deficits (back to work)*<br>*8) Improvement in psychiatric symptoms, F/U 15 mo, anterograde amnesia*

Bamford A, et al. Pediatric Herpes Simplex Virus Encephalitis Complicated by N-Methyl-D-aspartate Receptor Antibody Encephalitis. J Pediatric Infect Dis Soc. 2015 Jun;4(2):e17-21	*1*	*16 months*	*F*	*35*	***HSE**: lethargy, fever and focal seizures*<br><br>***AE**: encephalopathy, left hemiparesis, evolving right-sided movement disorder (orolingual and facial dyskinesia, dystonia, and right-sided ballismus)*	*NMDA (CSF, 1:50 and serum, 1:1000)*	*Right-sided dyskinesia and dystonia have reduced; however, left hemiparesis persists. There is severe global developmental impairment and she is fed through a gastrostomy feeding tube. The main benefit from treatment to date is improved awareness and social interaction, which has optimized neurorehabilitation potential in the setting of extensive acquired brain injury.*
Yushvayev-Cavalier Y, Nichter C, Ramirez-Zamora A. Possible autoimmune association between herpes simplex virus infection and subsequent anti-N-methyl-d-aspartate receptor encephalitis: a pediatric patient with abnormal movements. Pediatr Neurol. 2015 Apr;52(4):454-6.	*1*	*6 months*	*F*	*16*	***HSE**: fever, vomiting, watery diarrhoea, seizures* ***AE**: insomnia, irritability, altered level of consciousness, choreoathetoid movements and dysautonomia*	*NMDA (CSF)*	*At 3 months' follow-up, her abnormal movements were completely resolved and she was seizure-free. At 9 months' follow-up, she has global developmental delay but appears more social and interactive. She is tolerating a slow taper from clobazam and tetrabenazine, and valproate continues to be her primary antiepileptic therapy.*
Bektaş Ö et al. Anti-N-methyl-D-aspartate receptor encephalitis that developed after herpes encephalitis: a case report and literature review. Neuropediatrics. 2014 Dec;45(6):396-401.	*1*	*19 months*	*F*	*28*	***HSE**: seizures and loss of consciousness*<br><br>***AE**: choreoathetoid movements*	*NMDA (CSF and serum)*	*The patient's dyskinetic movements disappeared. She was aware of her environment, yet she was unable to speak. In addition, she was able to sit unsupported and crawl, but she remained unable to walk.*

Desena A et al. Herpes simplex encephalitis as a potential cause of anti-N-methyl-D-aspartate receptor antibody encephalitis: report of 2 cases. JAMA Neurol. 2014 Mar;71(3):344-6.	*1*	*"20s"*	*M*	*28*	***HSE**: headaches, fever, malaise and 1 week of confusion*<br><br>***AE**: speech declined and he began having behavioural changes.*	*NMDA (serum)*	*He was ambulatory and verbal and could process simple tasks. He had ongoing episodic outbursts.*
Mohammad SS et al. Herpes simplex encephalitis relapse with chorea is associated with autoantibodies to N-Methyl-D-aspartate receptor or dopamine-2 receptor. Mov Disord. 2014 Jan;29(1):117-22.	*2*	*1) 7*<br>*2) 12 months*	*1)M 2) F*	*1) 15*<br>*2) 42*	***HSE**: 1) Fever, encephalopathy, focal symptoms*<br>*2) Fever, encephalopathy, focal seizures*<br><br>***AE**: 1) Encephalopathy, chorea, dystonia, dysautonomia*<br>*2) Encephalopathy, chorea*	*1) NMDA (serum and CSF)*<br>*2) D2R (serum - no CSF for NMDA taken)*	*1) 9 months; developmental delay*<br>*2) 14 years; refractory epilepsy, intellectual disability, behavioural problems*
Wickström R et al. Viral triggering of anti-NMDA receptor encephalitis in a child - an important cause for disease relapse. Eur J Paediatr Neurol. 2014 Jul;18(4):543-6.	*1*	*11 months*	*F*	*15*	***HSE**: status epilepticus preceded by one day of high fever, vomiting and diarrhoea*<br><br>***AE**: fever and displayed a progressive change in behaviour that was perceived as aggressive in combination with oral and truncal choreoathetosis or dystonia*	*NMDA (serum 1:64 and CSF, 1:16)*	*At the age of 3 years, she has no apparent motor deficits but is hyperactive, delayed in her cognitive and speech development and with intractable epilepsy.*
Haddad A et al. Anti-NMDAR encephalitis following herpes simplex encephalitis: A case report and update on diagnostic and treatment. Rev Neurol (Paris). 2022 Dec;178(10):1107-1109.	*1*	*73*	*M*	*60*	***HSE**: Not discussed*<br><br>***AE**: fluctuations of consciousness quickly leading to coma, fever and respiratory failure.*	*NMDA (CSF)*	*Total recovery in the following years*

Supplementary Table 3 Investigations and treatment of post-HSE AE patients acquired from the literature search

Reference	CSF Constituents	EEG	MRI-brain	Biopsy	Treatment
**Ayvacioğlu Çağan C et al. Life After Tetra Hit: Anti-NMDAR Encephalitis After HSV Encephalitis in a NMOSD Coexistent with Sjögren's Syndrome. Noro Psikiyatr Ars. 2022 Apr 12;59(2):161-163.**	**HSE**: WCC – "mild lymphocytosis"<br>Protein – 47mg/dl<br>OCBs - -ve,<br>Cytology –mild lymphocytosis.<br>PCR/serology – HSV-1 IgG and HSV PCR +ve<br><br>**AE**: WCC – not disclosed<br>Protein – 102.1 mg/dL HSV PCR<br>PCR/serology – HSV-1 PCR -ve	**HSE**: PLEDS in the left fronto-temporal region<br><br>**AE**: Unknown	**HSE**: T2-hyperintense lesion with cytotoxic oedema in the left medial temporal, hippocampal and insular areas<br><br>**AE**: T2 hyperintensity and Gd enhancement in the T1W images on the left limbic area	Not performed	**HSE**: 21 days of aciclovir<br><br>**AE**: IVIg (0.4g/kg/day) for 5 days and continued weekly thereafter with additional rituximab

**Berek K, et al. Caspr2 antibodies in herpes simplex encephalitis: an extension of the spectrum of virus induced autoimmunity? - A case report. BMC Neurol. 2022 Apr 5;22(1):131.**	**HSE:** WCC – 8/ul<br>Protein – 0.73g/l<br>OCBs – not disclosed<br>PCR/serology - HSV-1 PCR +ve<br><br>**AE:** WCC – 65/ul<br>Protein – not disclosed<br>OCBs – pattern II<br>PCR/serology – HSV-1 PCR -ve	**HSE**: bilateral slowing with periodic lateralized discharges<br><br>**AE**: changes improved	**HSE:** T2-hyperintense swelling in the left temporo-mesial lobe, including cortical and subcortical areas, hippocampus and amygdala. Within these regions partially a diffusion-restriction of the cortex was found; in addition there was a thin contrast-enhancing rim subcortically at the lateral border of the T2-hyperintense swelling<br><br>**AE:** minor regression of the T2 hyperintense lesion in the left temporal lobe and a reduction of the left hippocampal volume	Not performed	

**Gomez et al 2021. The first recorded case of herpes simplex virus encephalitis followed by anti-NMDA receptor autoimmune encephalitis after resection of meningioma. J Interdisciplinary Neurosurgery; 24:1-3.**	**HSE:** WCC – 39/ul<br>Protein – 0.68g/l<br>OCBs – not disclosed<br>PCR/serology – HSV-1 PCR +ve<br><br>**AE:** "no remarkable differences between previous analyses; and PCR film array was negative for HSV-1"	Not discussed	**HSE:** T2-hyperintensities within both frontal lobes and gradient echo demonstrating intraparenchymal haemorrhage in the left insula and bifrontal lobes. Diffusion restriction within both straight gyri, both insulae, right cingulate gyrus.<br><br>**AE:** greater cerebral oedema and acute frontal intraparenchymal haemorrhage.	Right frontal lobe biopsy "confirmed HSE"	**HSE**: 21 days of aciclovir<br><br>**AE:** IVIg (0.4g/kg/day) for 5 days and "a new cycle of aciclovir was started" following biopsy results

**Hu S et al. HSV encephalitis triggered anti-NMDAR encephalitis: a case report. Neurol Sci. 2021 Mar;42(3):857-861.**	**HSE and AE:** WCC – 48/ul<br>Protein – "normal"<br>OCBs – not discussed<br>PCR/serology – HSV-1 PCR +ve	Diffuse slow waves, especially on the right temporal lobe	**HSE and AE:** T2 and DWI hyperintense lesions of the right temporal lobe	Not performed	**HSE**: 21 days of IV aciclovir<br>**AE**: IV methylprednisolone (1000mg/day) on d12, IVIg (0.4g/kg/day for 5 days) repeated after 7 days

**Jiang Y et al. Virus reactivation after immunotherapy of anti-NMDAR encephalitis secondary to herpes simplex encephalitis: A case report. Brain Dev. 2021 Nov;43(10):1057-1060.**	**HSE:** WCC – normal<br>Protein – normal<br>OCBs – not discussed<br>PCR/serology – HSV-1 PCR +ve<br><br>**AE**: WCC – normal<br>Protein – normal<br>OCBs – not discussed<br>PCR/serology – HSV-IgM and HSV-PCR -ve	Not discussed	**HSE:** FLAIR) sequence hyper-intensity signal in the right parietal lobe and temporal lobe on day 2. Multiple lesions in bilateral frontotemporal parietal lobes and right thalamus with right parietal cortex necrosis on day 9<br><br>**AE:** Abnormal signals were detected in bilateral temporal parietal lobes, right frontal lobe and thalamus, accompanied by right temporal parietal lobes and left parietal malacia lesions in MRI on day 36	Not performed	**HSE:** 21 days of IV aciclovir<br><br>**AE:** Methylprednisolone 20 mg/kg/day for 5 days followed by intravenous immunoglobulin. Prednisolone given after methylprednisolone treatment (1.5 mg/kg/d for 2 weeks, then reduced to 1 mg/kg/d and maintenance). Second-line therapy (rituximab, 375 mg/m2) two weeks later. Recurrent HSE necessitated another 21 days of IV aciclovir.

**Manglani M et al. Anti-NMDAR Encephalitis After Neonatal HSV-1 Infection in a Child With Low TLR-3 Function. Pediatrics. 2021 Sep;148(3):e2020035824.**	**HSE**: WCC – 118/ul<br>Protein – 0.69g/L<br>OCBs – not discussed<br>PCR/serology - HSV-1 PCR +ve<br><br>**AE**: WCC – 35/ul<br>Protein – 0.78g/L<br>OCBs – not discussed<br>PCR/serology – HSV-1 PCR -ve	Not discussed	**HSE**: bilateral temporal lobes, posterior inferior frontal lobes, left insula, left hippocampus, and left thalamus with concurrent mild leptomeningeal enhancement<br><br>**AE**: encephalomalacia and gliosis in the left mesial temporal lobe and left insular cortex		**HSE:** 21 days of IV aciclovir<br><br>**AE:** 5 days of 300 mg IV methylprednisolone, 4-day course of 5 g intravenous immunoglobulin (IVIG), and 5 rounds of plasma exchange (PLEX). 2nd line rituximab therapy was initiated. He received 4 doses of 190 mg rituximab over a 4-week period. The patient was discharged to rehabilitation on suppressive oral acyclovir treatment, IVIG infusions every 3 weeks, and rituximab infusions at 6-month intervals

**Swayne A et al. Analysing triggers for anti-NMDA-receptor encephalitis including herpes simplex virus encephalitis and ovarian teratoma: results from the Queensland Autoimmune Encephalitis cohort. Intern Med J. 2022 Nov;52(11):1943-1949.**	**HSE:** WCC 3-100/ul<br>Protein – 0.36g/L<br>OCBs – not discussed<br>PCR/serology – HSV-1 PCR +ve<br><br>**AE:** WCC – 20-35/ul<br>Protein – 0.72-1.4g/L<br>OCBs – not discussed<br>PCR/serology – HSV-1 PCR -ve	Not discussed	**HSE:** 100% abnormal - T2/FLAIR hyperintensity within the medial temporal lobe<br><br>**AE:** no progressive change after anti-NMDA relapse	Not performed	**HSE:** 21 days of IV aciclovir<br><br>**AE:** methylprednisolone (100%), IVIg (2/3), PLEX (1/3), rituximab (100%)

**Brás A et al. Anti-NMDAR Encephalitis Following Herpes Simplex Virus Encephalitis: 2 Cases From Portugal. Neurohospitalist. 2020 Apr;10(2):133-138.**	**1) HSE:** WCC –"inflammatory"<br>Protein – not discussed<br>OCBs – not discussed<br>PCR/serology – HSV-2 PCR +ve**;**<br>**AE:** WCC – not discussed<br>Protein – not discussed<br>OCBs – not discussed<br>PCR/serology HSV-2 PCR -ve<br>**2) HSE:** WCC – not discussed<br>Protein – not discussed<br>OCBs – not discussed<br>PCR/serology – HSV-1 PCR +ve<br>**AE:** WCC – not discussed<br>Protein – not discussed<br>OCBs – not discussed<br>PCR/serology – HSV-1 PCR -ve	Not discussed	1) **HSE:** left temporal lobe lesion compatible with acute HSV; temporal brain lesion was discreetly increased on the follow-up brain MRI (day 16)<br>**AE:** temporal brain lesion was discreetly<br>increased on the follow-up brain MRI (day 16)<br>2) **HSE:** a right temporal lobe lesion suggestive of acute encephalitis<br>**AE:** severe right hippocampus atrophy	Not performed	1) **HSE:** aciclovir and foscarnet later<br>**AE:** IV MP (5 days), IVIg (23g/d for 5d), oral prednisolone taper and IVIG over 8 months<br>2) **HSE:** aciclovir<br>**AE:** IV MP (5d), IVIg (25g/d for 5 days), oral prednisolone taper.

**Peters J, Wesley SF. Case of concurrent herpes simplex and autoimmune encephalitis. Neurol Neuroimmunol Neuroinflamm. 2020 Oct 2;7(6):e897.**	**HSE:** WCC – 0<br>Protein – 0.91g/dl<br>OCBs – not discussed<br>PCR/serology – HSV-1 PCR +ve<br>**AE:** WCC – 76/ul<br>Protein – 0.89g/dl<br>OCBs – not discussed<br>PCR/serology – HSV-1 PCR -ve	Continuous EEG monitoring showed intermittent nonconvulsive seizures arising from the left temporal lobe	**HSE:** FLAIR) hyperintensity of the medial right temporal lobe and right insular cortex with patchy diffusion restriction<br>**AE:** showed worsening right temporal lobe T2 FLAIR hyperintensity and haemorrhage into the right medial temporal lobe	Not performed	**HSE:** aciclovir<br>**AE:** IVIg (5d) then IV MP, 2 weeks later rituximab (all together with 21d aciclovir)

**Armangue T, et al. Toll-like receptor 3 deficiency in autoimmune encephalitis post-herpes simplex encephalitis. Neurol Neuroimmunol Neuroinflamm. 2019 Sep 5;6(6):e611.**	**HSE:** unable to perform acutely given raised intracranial pressure<br>**AE:** WCC – 15/ul<br>Protein – 1g/dl<br>OCBs – not discussed<br>PCR/serology – HSV-1 PCR +ve	Not discussed	**AE:** CT - new haemorrhagic temporal lesions requiring decompressive hemicraniectomy but no MR imaging reported	Positive HSV-1 PCR in brain tissue following decompressive hemicraniectomy	**HSE:** 3 weeks IV aciclovir **AE:** repeat aciclovir followed by oral valganciclovir; IV steroids, IVIg, RTX

**Handoko M et al. Autoimmune Glial Fibrillary Acidic Protein Astrocytopathy Following Herpes Simplex Virus Encephalitis in a Pediatric Patient. Pediatr Neurol. 2019 Sep;98:85-86.**	**HSE:** WCC – not discussed<br>Protein – not discussed<br>OCBs – not discussed<br>PCR/serology – HSV-1 PCR +ve<br>**AE:** WCC – normal<br>Protein – 0.97g/l<br>OCBs – not discussed<br>PCR/serology – HSV-1 PCR -ve	Not discussed	**HSE:** right greater than left temporal and frontal lobe swelling, suggestive of HSE **AE:** MRI showed sequelae of HSE without acute findings	Not performed	**HSE:** 3 weeks IV aciclovir **AE:** 5d IV MP and IVIg w/ oral prednisolone taper; relapsed -> monthly IVIg, MMF and extended oral steroids

**Yan Hung SK, Hiew FL, Viswanathan S. Anti-NMDAR Encephalitis in Association with Herpes Simplex Virus and Viral and Bacterial Zoonoses. Ann Indian Acad Neurol. 2019 Jan-Mar;22(1):102-103.**	**HSE/AE:** WCC – 0<br>Protein – 0.46g/l<br>OCBs – not discussed<br>PCR/serology – HSV-1 and Japanese encephalitis PCR +ve	Ictal focal discharges from the left frontocentral region with contralateral spread and after-going theta slowing	Brain MRI NAD	Not performed	IV aciclovir (unclear duration) AE: IVIg, then oral maintenance steroids and azathioprine. IV cyclophosphamide 500mg/m2 given later.

**Ko JM et al. Hyperammonemia in a case of herpes simplex and anti-N-methyl-d-aspartate receptor encephalitis. Brain Dev. 2019 Aug;41(7):634-637.**	**HSE:** WCC – 324/ul<br>Protein – 0.77g/l<br>OCBs – not discussed<br>PCR/serology – HSV-1 PCR +ve<br>**AE:** nad other than NMDA.	Not discussed	**HSE:** cortical swelling and increased signal intensity in the right temporo-occipital area<br>**AE:** encephalomalacia change of the right temporo-occipital area, but no new lesion was detected		**HSE:** 3 weeks IV aciclovir<br>**AE:** IVIg (5d 400mg/kg/d), methylprednisolone (3d; 30mg/kg/day), then no improvement hence RTX (375mg/m2 BSA weekly x 5 doses) and cyclophosphamide infusion (750mg/m2 BSA monthly x 5 doses)

**Mrad L et al. Severe presentation of antibody-negative, postinfectious steroid-responsive encephalitis and atonic bladder after herpes simplex encephalitis. BMJ Case Rep. 2019 Jul 22;12(7):e230005.**	**HSE:** WCC – 51/ul<br>Protein – not discussed<br>OCBs – not discussed<br>PCR/serology – HSV-1 PCR +ve<br>**AE:** WCC – 44/ul<br>Protein – 1.86g/l<br>OCBs – not discussed<br>PCR/serology – HSV-1 PCR -ve	Right-sided focal frontal temporal slowing with frequent moderate amplitude sharp waves. A right temporal seizure was also identified	Unable to obtain due to PPM	Not performed	**HSE:** aciclovir<br>**AE:** IV MP (5d of PO prednisolone), continued aciclovir then switched to valaciclovir

**Sahar N, Nurre AM, Simon RQ. Infectious Trigger for Autoimmune Encephalitis: A Case Report and Literature Review. Case Rep Infect Dis. 2019 Nov 6;2019:5731969.**	**HSE:** WCC – 25/ul<br>Protein – 0.94g/l<br>OCBs – not discussed<br>PCR/serology – HSV-1 PCR +ve<br>**AE:** WCC – "lymphocytic pleocytosis"<br>Protein – not discussed<br>OCBs – not discussed<br>PCR/serology – HSV-1 PCR -ve	Focal slowing in the right frontal/parietal regions without any epileptic discharges	**HSE:** oedema in the right frontotemporal lobes and left frontal lobe<br>**AE:** faint cortical enhancement in the right anterolateral temporal lobe	Not performed	**HSE:** 21d of aciclovir<br>**AE:** PLEX x 2 courses, RTX, then PLEX, steroids and cyclophosphamide. Further Rx with IVIg later.

**Alexopoulos H et al. Postherpes simplex encephalitis: a case series of viral-triggered autoimmunity, synaptic autoantibodies and response to therapy. Ther Adv Neurol Disord. 2018 Apr 23;11:1756286418768778.**	**HSE:** WCC – 1) 332/ul 2) 165/ul 3) 140/ul<br>Protein – not discussed<br>OCBs – not discussed<br>PCR/serology – 1-3) HSV-1 PCR +ve<br>**AE:** WCC – 1) not discussed 2) 185/ul 3) not discussed<br>Protein – 1) not discussed 2) 0.75g/l 3) not discussed<br>OCBs – 1-3)not discussed<br>PCR/serology – 1-3) HSV-1 PCR -ve	No abnormalities on patient 2 (none others revealed)	**HSE:** 1) Haemorrhagic lesion temporal lobe<br>2) Symmetrical focal lesions; temporal lobes and basal ganglia<br>3) Symmetrical focal lesions; temporal lobes and basal ganglia<br>**AE:** 1) dilatation of subarachnoid spaces with enlargement of the third and lateral ventricles the prior haemorrhagic lesion in the left temporal lobe related to HSV-1 infection remains now as a gliotic area 2) not discussed<br>3) gliosis and atrophy of the right temporal lobe, as previously observed	Not performed	**HSE:** All aciclovir 21 days **AE:** 1) IVIg, methylprednisolone IV (5d, 30mg/kg/d) -> PO prednisolone taper, then RTX<br>2) no immunosuppression<br>3) no immunosuppression

**Armangue T et al. Spanish Herpes Simplex Encephalitis Study Group. Frequency, symptoms, risk factors, and outcomes of autoimmune encephalitis after herpes simplex encephalitis: a prospective observational study and retrospective analysis. Lancet Neurol. 2018 Sep;17(9):760-772.**	**HSE/AE:** Not discussed in full detail but NSAb found 100% of CSF samples	Not discussed	**HSE/AE:** no significant FLAIR or DWI differences in volume lesion between patients who developed AE and those who did not.<br>**AE:** more likely to have necrosis with cystic lesions in MRIs obtained at follow-ups later than 4 months post-HSE.	Not performed	**HSE:** 21d aciclovir<br>**AE:** no treatment (7%, 13%), first line therapy 93%; 87%), 2nd line (56%,26%)

**Li J et al. Autoimmune GFAP astrocytopathy after viral encephalitis: A case report. Mult Scler Relat Disord. 2018 Apr;21:84-87.**	**HSE:** not discussed<br>**AE:** WCC – 34<br>Protein – 1g/l<br>OCBs – unpaired<br>PCR/serology – HSV-1 PCR -ve	Sharp and slow wave complexes emanating from the left temporal regions	**HSE:** T2/FLAIR hypersignal in the bilateral temporal lobe<br>**AE:** T2/FLAIR-hyperintense lesions in frontal and parietal white matter, associated with slight atrophy of the bilateral temporal lobe, contrast-enhanced brain MRI showed remarkable linear enhancement oriented radially to the ventricles	Not performed	**HSE:** aciclovir **AE:** intravenous dexamethasone followed by oral prednisone then MMF as steroid sparing

**Omae T et al. Cytokine/chemokine elevation during the transition phase from HSV encephalitis to autoimmune anti-NMDA receptor encephalitis. Brain Dev. 2018 Apr;40(4):361-365.**	**HSE:** WCC – 310/ul<br>Protein – 0.33g/l<br>OCBs – not discussed<br>PCR/serology – HSV-1 PCR +ve<br>**AE:** WCC – 67/ul<br>Protein – 0.67g/l<br>OCBs – not discussed<br>PCR/serology – HSV-1 PCR -ve	Not discussed	**HSE:** high signal in the left anterior, medial temporal regions<br>**AE:** high signal to the anterior-lateral areas adjacent to the initial HSV lesion, involving both gray and white matters	Not performed	**HSE:** aciclovir and IVIg (2g/kg) and Ara-A (15mg/kg).<br>**AE:** Methylprednisolone (30mg/kg/d for 3/7), IVIg, repeat IVIg and MP, PLEX followed by CYC (500mg/m2 monthly for 6/12), maintenance MMF (600mg/m2/day) for 1 year, 2nd CYC pulse

**Kothur K et al. Cerebrospinal fluid cyto-/chemokine profile during acute herpes simplex virus induced anti-N-methyl-d-aspartate receptor encephalitis and in chronic neurological sequelae. Dev Med Child Neurol. 2017 Aug;59(8):806-814.**	**HSE**: WCC – 7/ul<br>Protein – 0.85g/l<br>OCBs – not discussed<br>PCR/serology – HSV-1 PCR +ve<br>**AE:** WCC – pleocytosis "declined"<br>Protein – not discussed<br>OCBs – not discussed<br>PCR/serology – HSV-1 PCR +ve	Not discussed	**HSE:** high signal in the right temporal, frontal lobe, and insular regions<br>**AE:** worsening high signal in the right temporal and occipital white matter without any new areas of necrosis	Not performed	**HSE:** aciclovir<br>**AE:** methylprednisolone (d15, one dose), IV MP (3d, 30mg/kg/d), IVIg, RTX (weekly 375mg/m2 for 4 weeks), oral prednisolone taper, monthly IVIg for 6 months.

**Nosadini M et al. Herpes simplex virus-induced anti-N-methyl-d-aspartate receptor encephalitis: a systematic literature review with analysis of 43 cases. Dev Med Child Neurol. 2017 Aug;59(8):796-805.**	**HSE:** WCC – 10/ul<br>Protein – 0.38g/l<br>OCBs – not discussed<br>PCR/serology – HSV-1 PCR +ve<br>**AE:** WCC – 39/ul<br>Protein – 0.97g/l<br>OCBs – not discussed<br>PCR/serology – HSV-1 PCR -ve	Diffuse encephalopathy	**HSE:** asymmetrical abnormal signal intensity involving bilateral frontal, precentral and central subcortical white matter, deep gray and white matter, bilateral ventrolateral thalami, and left midbrain and pons along the corticospinal tracts<br>**AE:** evolution of the previous lesions, in the absence of new areas of cytotoxic oedema	Not performed	**HSE:** aciclovir<br>**AE:** IV MP (3d) then PO prednisolone

**Strippel C et al. Treating refractory post-herpetic anti-N-methyl-d-aspartate receptor encephalitis with rituximab. Oxf Med Case Reports. 2017 Jul 3;2017(7):omx034.**	**HSE:** WCC – 3/ul<br>Protein – 0.793g/l<br>OCBs – type 3<br>PCR/serology – HSV-1 PCR +ve<br>**AE:** WCC – 2/ul<br>Protein – 0.433g/l<br>OCBs – type 3<br>PCR/serology – HSV-1 PCR -ve	Not discussed	**HSE:** right more than left temporal scarring before initiation of rituximab (left panel)<br>**AE:** follow-up MRI (coronal FLAIR) showing progressive atrophy involving both temporal lobes	Not performed	**HSE:** aciclovir **AE:** MP, PLEX and protein A immunoadsorption then repeat at 2 weeks with RTX

**Geoghegan S et al. Anti-N-Methyl-D-Aspartate Receptor Antibody Mediated Neurologic Relapse Post Herpes Simplex Encephalitis: A Case Series. Pediatr Infect Dis J. 2016 Aug;35(8):e258-61.**	**HSE:** WCC – 1) 6/ul 2) 373/ul 3) 51/ul<br>Protein – 1) 0.19g/l 2) 0.317g/l 3) 0.317g/l<br>OCBs – not discussed<br>PCR/serology – 1-3) HSV-1 PCR +ve<br>**AE:** WCC – 1) 25/ul 2) 51/ul 3) 47/ul<br>Protein – 1-3) not discussed<br>OCBs – not discussed<br>PCR/serology – 1) HSV-1 PCR -ve 2) HSV-1 PCR +ve 3) HSV-1 PCR -ve	Not discussed	**HSE:** 1) CT brain only (NAD)<br>2) and 3) consistent with encephalitis<br>**AE:** 1) bilateral temporoparietal and minor right thalamic haemorrhages with oedema<br>2) no changes<br>3) persistent bilateral frontal lobe and left perisylvian restricted diffusion	Not performed	**HSE:** 1) aciclovir **AE:** 1) methylprednisolone (30 mg/kg) were recommenced, and IVIG replacement (400 mg/kg/d ×5) was started w/ maintenance IVIg replacement w/ hypogammaglobulinaemia<br>2) oral prednisolone (2mg/kg) then IVIg<br>3) methylprednisolone (30mg/kg) and IVIg (400mg/kg/d for 5 days), PLEX (discontinued given autonomic instability) then IV RTX (375mg/m2 2 weeks apart)

**Morris NA et al. HSV encephalitis-induced anti-NMDAR encephalitis in a 67-year-old woman: report of a case and review of the literature. J Neurovirol. 2016 Feb;22(1):33-7.**	**HSE:** WCC – 730<br>Protein – 1.64g/l<br>OCBs – not discussed<br>PCR/serology – HSV-1 PCR +ve<br>**AE:** WCC – 10<br>Protein – 0.89g/l<br>OCBs – two OCBs<br>PCR/serology – HSV-1 PCR -ve	Right frontal lateral periodic discharges	**HSE**: increased T2/fluid attenuation inversion recovery<br>(FLAIR) intensity in the right temporal lobe,<br>medial/inferior<br>frontal lobe, and insula, with restricted diffusion in the right<br>temporal lobe and insula<br>**AE:** Repeat MRI showed gliosis in the same distribution of the right frontotemporal areas originally affected	Not performed	**HSE:** aciclovir(21d)<br>**AE:** IVIg (5d) then RTX (1000mg IV twice, 2 weeks apart) then CYC (750mg/m2 added for 3 months given catatonia

**Sutcu M et al. Role of Autoantibodies to N-Methyl-d-Aspartate (NMDA) Receptor in Relapsing Herpes Simplex Encephalitis: A Retrospective, One-Center Experience. J Child Neurol. 2016 Mar;31(3):345-50.**	**HSE:** WCC – 1) "mild pleocytosis" 2) "no pleocytosis"<br>Protein – 1) 0.82g/l 2) 0.6g/l<br>OCBs – not discussed<br>PCR/serology – HSV-1 PCR +ve<br>**AE:** not specified but HSV PCR now negative	**AE:** 1) high-amplitude rhythmic spike and waves over the right central and temporal areas 2) delta brushes with a beta activity of 15 to 25 Hz over the frontal lobes	**HSE:** 1) hyperintense lesion on right temporal lobe and thalamus<br>2) hyperintense lesion on left parietotemporal lobe, left precentral and postcentral gyrus.<br>**AE:** 1) no new changes<br>2) new hyperintense lesions on T2-weighted images of the bilateral thalamus extending into the bilateral cerebral peduncles and mesencephalon with haemorrhagic changes	Not performed	**HSE:** 1) 14d aciclovir x2 2) 21d aciclovir **AE:** 1) aciclovir only w/o improvement<br>2) IVIg (1g/kg for 2 d) and steroids (2mg/kg/d PO)

**Armangue T et al. Spanish Prospective Multicentric Study of Autoimmunity in Herpes Simplex Encephalitis. Autoimmune post-herpes simplex encephalitis of adults and teenagers. Neurology. 2015 Nov 17;85(20):1736-43.**	**HSE:** WCC – 1) not discussed 2) 2/ul 3) 110/ul 4) 239/ul 5) 460/ul 6) 32/ul 7) 49/ul 8) 250/ul<br>Protein – 1) not discussed 2) 0.5g/l 3) 0.74g/l 4) 0.66g/l 5) 0.51g/l 6) "normal" 7) 0.6g/l 8) 0.62g/l<br>OCBs – not discussed<br>PCR/serology – 1-8)HSV-1 PCR ve<br>**AE:** WCC – 1) 7/ul 2) 0/ul 3) 10/ul 4) 15/ul 5) 10/ul 6) 0/ul 7) 2/ul 8) 10/ul<br>Protein – 1) "normal" 2) "normal" 3) "normal" 4) 0.88g/l 5) 0.65g/l 6) "normal" 7) 1.12g/l 8)"normal"<br>OCBs – 1) not discussed 2) not discussed 3) unmatched 4) not discussed 5) not discussed 6) not discussed 7) not discussed 8) unmatched<br>PCR/serology –1-8) HSV-1 PCR -ve	Not discussed	**HSE:** 1) extensive left frontotemporal and mild right frontobasal T2 and FLAIR abnormalities, with mass effect and restricted diffusion<br>2) bilateral right greater than left frontotemporal T2-FLAIR hyperintensities with ADC restriction<br>3) left temporal T2/FLAIR hyperintensities with ADC restriction and mild contrast enhancement suggestive of HSE encephalitis<br>4) right greater than left temporal and hippocampal T2/FLAIR hyperintensities with ADC restriction without contrast enhancement<br>5) left temporal and hippocampal T2/FLAIR hyperintensities with ADC restriction<br>6) left temporal and hippocampal T2/FLAIR hyperintensities without ADC restriction but with intense contrast enhancement<br>7) no comparison MR<br>8) bilateral T2/FLAIR abnormalities without contrast enhancement in the temporal lobes, right greater left<br>**AE**:1)encephalomalacia in the previous areas of viral involvement without new necrotic regions but with expansion of the surrounding white matter changes<br>2) encephalomalacia of the previous right frontotemporal viral involvement, no new necrotic lesions, but mild	Not performed	**HSE:** 1) 21d aciclovir<br>2) 21d aciclovir<br>3) 21d aciclovir<br>4) 14d +14d on relapse aciclovir<br>5) 14d aciclovir<br>6) 21d aciclovir<br>7) 14d + 21 on relapse aciclovir<br>8) 15d aciclovir **AE:** 1) 5d IV MP<br>2) IVIg, IV MP followed my PO methylprednisolone<br>3) no immunosuppressants<br>4) IV corticosteroids, IVIg, RTX and CYC<br>5) IV MP<br>6) IV MP, IVIg and PLEX then RTX<br>7) IV MP<br>8) 5d IV MP (the 2 further monthly cycles)

			worsening of T2/FLAIR white matter changes bilaterally affecting the frontal lobes plus contrast enhancement<br>3) slight progression of the T2/FLAIR hyperintensities in the left temporal lobe with intense contrast enhancement and new involvement of the right temporal lobe<br>4) mild progression of the previous T2/FLAIR white matter bilateral temporal lobe hyperintensities with new intense contrast enhancement<br>5) mild expansion of the T2/FLAIR white matter changes in the left temporal lobe<br>6) not discussed<br>7) extensive bilateral right greater than left frontotemporal T2/FLAIR hyperintensities<br>8) slight progression of T2/FLAIR hyperintensity in the temporal lobes with new contrast enhancement		

**Bamford A et al. Pediatric Herpes Simplex Virus Encephalitis Complicated by N-Methyl-D-aspartate Receptor Antibody Encephalitis. J Pediatric Infect Dis Soc. 2015 Jun;4(2):e17-21.**	**HSE:** WCC – 12/ul<br>Protein – 0.76g/l<br>OCBs – not discussed<br>PCR/serology – HSV-1 PCR +ve<br>**AE:** not discussed	EEG: NAD	**HSE:** CT: extensive R-sided infarction<br>**AE:** MRI scan of the brain demonstrated extensive right-sided encephalomalacia with complete destruction of the parietal and temporal lobes	Not performed	**HSE:** aciclovir (750mg/m2->1500mg/m2)<br>**AE:** PLEX (5 cycles) x2

**Yushvayev-Cavalier Y et al. Possible autoimmune association between herpes simplex virus infection and subsequent anti-N-methyl-d-aspartate receptor encephalitis: a pediatric patient with abnormal movements. Pediatr Neurol. 2015 Apr;52(4):454-6.**	**HSE:** +ve HSV1 PCR<br>**AE:** "mildly elevated lymphocytic pleocytosis" and -ve HSV1 PCR	**EEG**: left frontotemporal slowing superimposed on generalised slowing	**HSE:** extensive bi-hemispheric regions of diffusion restriction involving the temporal, parietal and occipital lobes, which worse in the right hemisphere.<br>**AE:** no new abnormalities	Not performed	**HSE:** 21d aciclovir **AE:** RTX (375mg/m2) weekly for 4 weeks, CYC (500mg/m2) monthly for 6 months

**Bektaş Ö et al. Anti-N-methyl-D-aspartate receptor encephalitis that developed after herpes encephalitis: a case report and literature review. Neuropediatrics. 2014 Dec;45(6):396-401.**	**HSE:** WCC – 1/ul<br>Protein – 0.54g/l<br>OCBs – not discussed<br>PCR/serology – HSV-1 PCR +ve<br>**AE:** no comment of differential	Not discussed	**HSE:** hyperintense lesions (T2 + FLAIR) in the bilateral insular cortices, perisylvian area, and the temporoparietal area, and less obvious in the bilateral thalami. Restricted diffusion in the same regions.<br>**AE:** atrophy in the opercular area	Not performed	**HSE**: 21d aciclovir and steroids<br>**AE:** IV MP, IVIg (400mg/kg/d for 5d) x2, PLEX 3x/week for 3/52, then RTX 375mg/m2 weekly (total = 12 doses and monthly CYC (500mg/m2, total 6 doses)

**Desena A et al. Herpes simplex encephalitis as a potential cause of anti-N-methyl-D-aspartate receptor antibody encephalitis: report of 2 cases. JAMA Neurol. 2014 Mar;71(3):344-6.**	**HSE:** WCC – 128/ul<br>Protein – 1.3g/l<br>OCBs – not discussed<br>PCR/serology – HSV-1 PCR +ve<br>**AE:** WCC – 128/ul<br>Protein – 1.3g/l<br>OCBs – not discussed<br>PCR/serology – HSV-1 PCR -ve	Not discussed	**HSE:** bitemporal oedematous lesions, greater on the left than the right sides **AE:** chronic bifrontal and temporal damage consistent with his prior HSV infection	Not performed	**HSE:** 21d aciclovir<br>**AE:** PLEX, IVIg (2g/kg over 5d) then CYC 1g/m2 repeated monthly

**Mohammad SS et al. Herpes simplex encephalitis relapse with chorea is associated with autoantibodies to N-Methyl-D-aspartate receptor or dopamine-2 receptor. Mov Disord. 2014 Jan;29(1):117-22.**	**HSE/AE –** no breakdown	1) left temporal lobe epileptic activity with slowing<br>2) not discussed	**HSE:** 1) right temporal lobe changes<br>2) hyperintensity in the left temporal lobe cortex and white matter<br>**AE:** 1) persisting right temporal lobe changes with symmetric hyperintensity in both putamen<br>2) not discussed	2) Left temporal lobectomy revealed destructive architecture, cystic changes, foamy macrophages, extensive gliosis, and neuronal loss. Perivascular lymphocytic aggregates demonstrating chronic active encephalitis. HSV1 testing on the brain tissue using viral culture and PCR was negative.	**HSE:** 1) aciclovir 14d<br>2)21d aciclovir<br>**AE:** 1) IV steroids, 2g/kg IVIg then CYC 3 doses 750mg/m2<br>2) PO prednisolone (2mg/kg/d for 1/12) and IVIg 2g/kg

**Wickström R et al. Viral triggering of anti-NMDA receptor encephalitis in a child - an important cause for disease relapse. Eur J Paediatr Neurol. 2014 Jul;18(4):543-6.**	**HSE:** +ve HSV PCR - no breakdown<br>**AE:** WCC – 30/ul<br>Protein – not discussed<br>OCBs – not discussed<br>PCR/serology – HSV-1 PCR -ve	Delta activity over left temporal lobe then 15-20Hz bi-hemispheric activity	**HSE:** CT normal<br>**AE:** CT revealed left temporal necrosis	Not performed	**HSE:** aciclovir 21d<br>**AE:** IVIg (1g/kg for 2/7), prednisolone (2mg/kg/d)
**Haddad A et al. Anti-NMDAR encephalitis following herpes simplex encephalitis: A case report and update on diagnostic and treatment. Rev Neurol (Paris). 2022 Dec;178(10):1107-1109.**	**HSE:** not discussed<br>**AE:** WCC – not discussed<br>Protein – 1g/l<br>OCBs – not discussed<br>PCR/serology – HSV-1 PCR -ve	Not discussed	**HSE:** MRI typical<br>**AE:** gadolinium enhancement in the right polar temporal lobe in the area of previous HSE	Not performed	**HSE:** aciclovir (unclear duration);<br>**AE:** 5 days IVIg, 3 months steroids, 3 injections of RTX

HSE, herpes simplex virus encephalitis; AE, autoimmune encephalitis; PCR, polymerase chain reaction; Ig, immunoglobulin; IVIg, intravenous immunoglobulin; RTX, rituximab; PLEX, plasma exchange; OCBs, oligoclonal bands; WCC, white cell count

Supplementary Table 4 Post-HSE AE publications with negative NSAbs or NSAbs with unknown antigenic targets.

Demographic	Clinical presentation	Serum NSAb	CSF NSAb	Immunotherapy	Time from HSE to AE (days)	Outcome	Publication
**6yo F**	Severe headache, encephalopathy, raised ICP (requiring decompressive craniectomy) and behavioural changes	Not discussed	NSAb positive (titre not discussed but strong rat hippocampal neurone reactivity)	IV steroids, IVIg, RTX	510	"Neurological improvement"	[8]
**75yo F**	Encephalopathy and generalised choreiform movements	Antibody negative	Antibody negative	IV MP	28	3 months follow-up: progressive improvement including a MOCA improving to 26/30 compared with 14/30	[9]
**Median age, 56 (4.5 months - 80 years); n= 19; M:F, 0.7:1**	Change behaviour 68%, seizures 47%, Encephalopathy 53%, movement disorder 21%, dysautonomia 0%	3 NSAb positive (titre, 1:400-1:800 from 2 patients)	15 NSAb positive (titre range, 1:5 - 1:160) (4 serum/CSF double NSAb negative patients)	No treatment 11%, first line therapy 95%, 2nd line 21%	Median 31 (11- 255)	Median mRS 3 (2-5)	[3]
**Median age, 56 (29 - 69); n=3; M:F, 0.7:1**	Change in behaviour 66%, seizures 66%, Encephalopathy 66%, movement disorder 0%, dysautonomia 0%	1 NSAb positive (no titres)	3 NSAb positive (no titres)	First line therapy 100%, second line therapy 33%	Median 21 (12-30)	**Patient 1** (3 months follow-up): mild aphasia<br>**Patient 2** (12 months follow-up): minor deficits and back to work<br>**Patient 3** (15 months follow-up): improvement in psychiatric symptoms but with ongoing anterograde amnesia	[5]

NSAb positivity assumes autoreactivity with an unknown antigen on immunohistochemistry. Negative NSAb included if a compatible clinical phenotype with secondary AE is demonstrated. NSAb, neuroglial surface antibodies; mRS, modified Rankin score; MP, methylprednisolone; IVIg, intravenous immunoglobulin; RTX, rituximab

References

[1] Dalmau J, Tüzün E, Wu HY, et al. Paraneoplastic anti–N-methyl-D-aspartate receptor encephalitis associated with ovarian teratoma. Ann Neurol 2007;61:25–36. https://doi.org/10.1002/ANA.21050.

[2] Venkatesan A, Tunkel AR, Bloch KC, et al. Case definitions, diagnostic algorithms, and priorities in encephalitis: consensus statement of the international encephalitis consortium. Clin Infect Dis 2013;57:1114–28. https://doi.org/10.1093/CID/CIT458.

[3] Armangue T, Spatola M, Vlagea A, et al. Frequency, symptoms, risk factors, and outcomes of autoimmune encephalitis after herpes simplex encephalitis: a prospective observational study and retrospective analysis. Lancet Neurol 2018;17:760–72. https://doi.org/10.1016/S1474-4422(18)30244-8.

[4] Nosadini M, Mohammad SS, Corazza F, et al. Herpes simplex virus-induced anti-N-methyl-d-aspartate receptor encephalitis: a systematic literature review with analysis of 43 cases. Dev Med Child Neurol 2017;59:796–805. https://doi.org/10.1111/DMCN.13448.

[5] Armangue T, Moris G, Cantarín-Extremera V, et al. Autoimmune post-herpes simplex encephalitis of adults and teenagers. Neurology 2015;85:1736–43. https://doi.org/10.1212/WNL.0000000000002125.

[6] Hargrave DR, Webb DW. Movement disorders in association with herpes simplex virus encephalitis in children: A review. Dev Med Child Neurol 1998;40:640–2. https://doi.org/10.1111/J.1469-8749.1998.TB15431.X.

[7] Brás A, André A, Sá L, et al. Anti-NMDAR Encephalitis Following Herpes Simplex Virus Encephalitis: 2 Cases From Portugal. Https://DoiOrg/101177/1941874419870987 2019;10:133–8. https://doi.org/10.1177/1941874419870987.

[8] Armangue T, Baucells BJ, Vlagea A, et al. Toll-like receptor 3 deficiency in autoimmune encephalitis post–herpes simplex encephalitis. Neurol Neuroimmunol Neuroinflammation 2019;6:611. https://doi.org/10.1212/NXI.0000000000000611.

[9] Mrad L, Moustakas A, Fuino R, et al. Severe presentation of antibody-negative, postinfectious steroid-responsive encephalitis and atonic bladder after herpes simplex encephalitis. BMJ Case Rep 2019;12. https://doi.org/10.1136/BCR-2019-230005.
